# Non-redundant implicational base of formal context with constraints using SAT

**DOI:** 10.7717/peerj-cs.1806

**Published:** 2024-01-31

**Authors:** Taufiq Hidayat, Asmala Ahmad, Hea Choon Ngo

**Affiliations:** 1 Faculty of Information and Communication Technology, Universiti Teknikal Malaysia Melaka, Melaka, Malaysia; 2 Informatics Department, Universitas Islam Indonesia, Yogyakarta, Indonesia

**Keywords:** Implicational base, Formal context, SAT problem, Attribute implication, Formal concept analysis

## Abstract

An implicational base is knowledge extracted from a formal context. The implicational base of a formal context consists of attribute implications which are sound, complete, and non-redundant regarding to the formal context. Non-redundant means that each attribute implication in the implication base cannot be inferred from the others. However, sometimes some attribute implications in the implication base can be inferred from the others together with a prior knowledge. Regarding knowledge discovery, such attribute implications should be not considered as new knowledge and ignored from the implicational base. In other words, such attribute implications are redundant based on prior knowledge. One sort of prior knowledge is a set of constraints that restricts some attributes in data. In formal context, constraints restrict some attributes of objects in the formal context. This article proposes a method to generate non-redundant implication base of a formal context with some constraints which restricting the formal context. In this case, non-redundant implicational base means that the implicational base does not contain all attribute implications which can be inferred from the others together with information of the constraints. This article also proposes a formulation to check the redundant attribute implications and encoding the problem into satisfiability (SAT) problem such that the problem can be solved by SAT Solver, a software which can solve a SAT problem. After implementation, an experiment shows that the proposed method is able to check the redundant attribute implication and generates a non-redundant implicational base of formal context with constraints.

## Introduction

**Figure 1:**
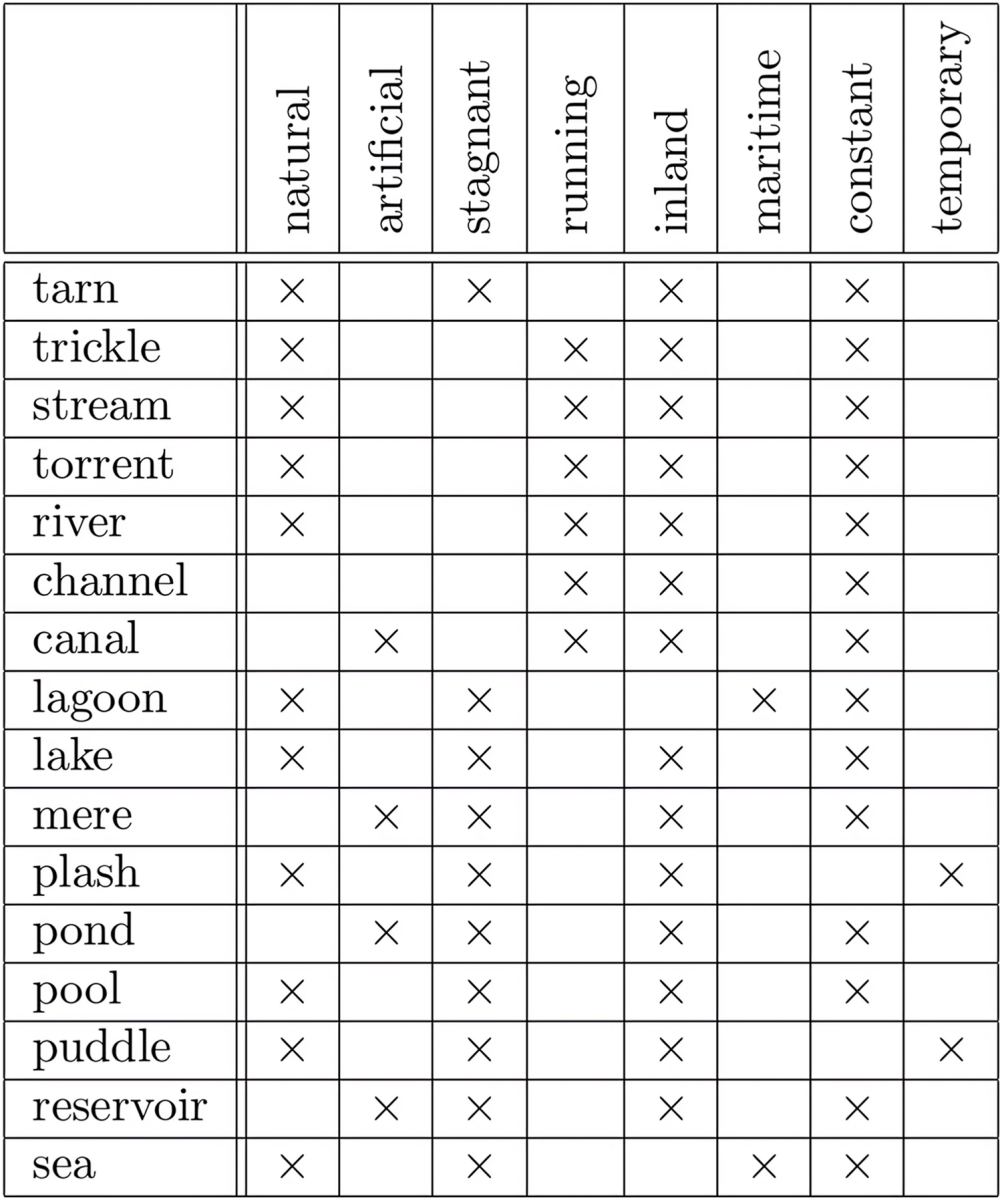
Formal context of “bodies of water” (Wille, 2005).

Formal context is a simple data type representing data. A formal context consists of a set of objects, a set of attributes, and a relation between both sets. The relation shows which attributes belong to each object. Visually, a formal context can be represented by a cross table where rows represent objects, columns represent attributes, and cells represent the relation (Ganter & Wille, 1999; Wille, 2005; Škopljanac Mačina & Blašković, 2014; Rocco, Hernandez-Perdomo & Mun, 2020; Bhuyan, Karmakar & Hazarika, 2018). Figure 1 is an example of formal context represented by a cross table.

Formal concept analysis (FCA) studies how to extract knowledge from a formal context and has been applied to many areas of data since a formal context is capable to represent any kinds of data. Some research has been conducted to extract knowledge from any data which is formulated in a formal context (Moulahi, 2021; Xu et al., 2019; Marín et al., 2021; Gély et al., 2022; Yan & Li, 2022; Zou et al., 2020; Janostik & Konecny, 2020; Atencia et al., 2020; Kötters & Eklund, 2020; Rocco, Hernandez-Perdomo & Mun, 2020; Kumar Mishra, Joshi & Mathur, 2020; Albahli & Melton, 2016). Therefore, formal concept analysis has been considered to be a method in knowledge discovery (Kumar, 2011).

Furthermore, formal concept analysis is promising method in knowledge discovery. Some research of application of formal concept analysis includes knowledge extraction, knowledge representation, and using of extracted knowledge. As a method in knowledge discovery, application of formal concept analysis and formal context covers many research domains including computer science and other domains. In computer science, some studies were successful to apply formal concept analysis for solving some problems in many sub-domains, *e.g*., datamining (Aragón, Medina & Ramírez-Poussa, 2022; Hao et al., 2023), machine learning (Janostik, Konecny & Krajča, 2022), data science (Bazin et al., 2022), intelligent system (Shao et al., 2023), information retrieval (Ojeda-Hernández, López-Rodríguez & Mora, 2023; Khattak et al., 2021), natural language processing (Marín et al., 2021; Jain, Seeja & Jindal, 2020), decision support system (Wei et al., 2020), recommendation system (Liu et al., 2022), semantic web (Jindal, Seeja & Jain, 2020), cloud computing (Khemili, Hajlaoui & Omri, 2022), data structure (Ferré & Cellier, 2020), mobile application (Kwon et al., 2021), software engineering (Carbonnel et al., 2020), and robotic (Zhang et al., 2023). In addition, some successful studies to apply formal concept analysis were in other domains, *e.g*., engineering (Rocco, Hernandez-Perdomo & Mun, 2020), mathematics (Jäkel & Schmidt, 2022; Rocco, Hernandez-Perdomo & Mun, 2020), biology (Gély et al., 2022), psychology (Belohlavek & Mikula, 2022), medicine (Md Saleh, Ab Ghani & Jilani, 2022), business (Wajnberg et al., 2018; Ravi, Ravi & Prasad, 2017; Acharjya & Das, 2017), and social science (Lang & Yao, 2023; Hao et al., 2021; Gao et al., 2021).

Implicational base is a kind of knowledge generated from formal context (Wille, 2005; Hidayat, bin Ahmad & Ishak bin Desa, 2021; Škopljanac Mačina & Blašković, 2014; Ganter & Obiedkov, 2016). Implicational base of a formal context is a set of attribute implications which are sound, complete, and non-redundant. Sound means that all of the attribute implications holds the formal context. Complete means that any attribute implications, which also hold the formal context, can be inferred from some attribute implications in the set. Non-redundant means that there is no attribute implication in the set which can be inferred from the others. Attribute implication is knowledge in the form of rule showing attribute dependencies. Some research in application of formal concept analysis extracted knowledge in this form Baixeries et al. (2018), Wei et al. (2020) and Dubois et al. (2021).

Recently, reducing generated knowledge for increasing its quality is concerned in formal concept analysis. Moreover, the size of the knowledge is sometime very large (Mouakher & Ben Yahia, 2019; Kuznetsov & Makhalova, 2018). The objective of this concern is to obtain interesting knowledge only. Some studies used prior knowledge to achieve it Pang et al. (2023), Zou et al. (2020), Ch, Dias & Vieira (2015). The prior knowledge is used as background knowledge in the process of formal concept analysis. Several studies used background knowledge to remove redundant knowledge which can be inferred from the background knowledge (Hidayat, bin Ahmad & Ishak bin Desa, 2021; Krishnan & Cherukuri, 2019; Sumangali & Kumar, 2019; Stumme, 1996; Belohlávek & Vychodil, 2008a; Viaud et al., 2016). An example of this study is to generate non-redundant implicational base (Hidayat, bin Ahmad & Ishak bin Desa, 2021; Hidayat, 2005). In the non-redundant implicational base, some attribute implications in an implicational base are ignored if they can be inferred from some other attribute implications together with the background knowledge (Hidayat, bin Ahmad & Ishak bin Desa, 2021; Hidayat, 2005). In Hidayat, bin Ahmad & Ishak bin Desa (2021), the problem to check whether an attribute implication is implied by some other attribute implications together with background knowledge is called a background-inferring problem.

A constraint is another form of prior knowledge which will cause redundant knowledge. A constraint is restriction of data and the data has to satisfy the constraint. In case of formal context, a constraint restricts some attribute-values of a formal context. An example of constraint in formal concept analysis is attribute dependency (Belohlávek, Sklenar & Zacpal, 2004; Belohlávek & Sklenar, 2005; Belohlávek & Vychodil, 2008b) where values of some attributes depend on another or some others. Let a formal context satisfy some constraints. This implies that information of the constraints will exist in the formal context. Unfortunately, the information will appear in an implicational base as generated knowledge in formal concept analysis. In other words, the implicational base will contain some attribute implications which can be inferred from the others together with information of the constraints. The attribute implications can be considered as redundant attribute implications based on the constraints.

To improve the quality of implicational base, it is necessary to remove the kind of redundant attribute implications. Thus, the implicational base becomes non-redundant based on some constraints. The important problem in this case is to check whether an attribute implication is redundant. In this article, it will be called constraint-inferring problem.

The next problem is how to solve the constraint-inferring problem. It is very important in implementation to solve the problem. An alternative solution is to encode the problem into satisfiability problem (SAT problem) such that it can be solved by the SAT solver, a specific software to solve the SAT problem. Recently, many SAT solvers can solve SAT problems with a large number of both clauses and variables in reasonable time. In Hidayat, bin Ahmad & Ishak bin Desa (2021) the background-inferring problem is successfully encoded into SAT (satisfiability) problem. The SAT problem is an interesting problem in computer science which is NP-complete (Biere et al., 2009). Many studies concerning this area have been conducted (Sohanghpurwala, Hassan & Athanas, 2017) where some of the studies do not only concern in the theoretical aspect but also in implementation and application (Ojeda, 2023; Zha, Chang & Noda, 2022; Alonso, Sánchez & Sánchez-Rubio, 2022; Ramamoorthy & Jayagowri, 2021; Mayank & Mondal, 2020). Some algorithms and some SAT solvers have been developed to solve the SAT problem (Fu et al., 2022; Berend, Golan & Twitto, 2022; Bian et al., 2020; Li et al., 2020; Molnár et al., 2020).

This article will propose a method to generate a non-redundant implicational base of formal context together with some constraints using SAT. This article will also propose a formulation of constraint which is suitable for any constraints and formal contexts such that it will be easy to define a constraint-inferring problem and to encode the problem into SAT problem. The proposed method will use a SAT solver to solve the SAT problem.

## Foundation

### Formal context

We will define some terminologies related to formal context. For the definition of formal context, we rewrite some definitions from our previous works in Hidayat, bin Ahmad & Ishak bin Desa (2021). A formal context is defined as triple $(G,M,I)$ which represents a finite set of objects *G*, a finite set of attributes *M*, and a relation *I* between *G* and *M*. The relation *I* shows some attributes belonging to each object.

**Definition 1.** *A formal context is defined as a triple* $(G,M,I)$ *which consists of two non-empty sets G and M, and a relation* $I \subseteq G \times M$. *G is a set of objects, whereas M is a set of attributes. For* $g \in G$ *and* $m \in M$, $(g,m) \in I$ or $gIm$ *means that the object* $g$ *has the attribute* $m$ (Ganter & Wille, 1999; Wille, 2005; Škopljanac Mačina & Blašković, 2014).

A cross table can represent a formal context $(G,M,I)$, with rows representing *G* and columns representing *M*. A cell of the table in row $g$ and column $m$ represents a relation *I* of object $g \in G$ and attribute $m \in M$. We cross the cell if $(g,m) \in I$. Recall Fig. 1. The figure shows a formal context of “bodies of water” (Wille, 2005).

**Definition 2.** *If* $A \subseteq G$ *is a set of objects of a formal context* $(G,M,I)$, *then Ganter & Wille (1999)*, Wille (2005), Škopljanac Mačina & Blašković (2014):

(1) $${A^I} = \{ m\mid (g,m) \in I,\forall g \in A\}$$

*Reversely, if* $B \subseteq M$ *is a set of attributes, then:*

(2) $${B^I} = \{ g\mid (g,m) \in I,\forall m \in B\}$$

Notation ${A^{II}}$ refers to ${({A^I})^I}$.

The symbol *I* in ${(.)^I}$ refers to *I* in the formal context $(G,M,I)$. If $A \subseteq G$ then ${A^I}$ means “all attributes that belong to all objects in *A*”. If $B \subseteq M$ then ${B^I}$ means that “all objects that have all attributes in *B*”.

**Definition 3.** *A many-valued context is a quadruple* $(G,M,W,I)$ *which consists of a set of objects G, a set of attributes M, a set of attribute values W, and a ternary relation* $I \subseteq G \times M \times W$ *where* $(g,m,w) \in I$ *and* $(g,m,v) \in I$ *implies* $w = v$ (Ganter & Wille, 1999; Ganter, 1996; Hidayat, 2005).

A triple $(g,m,w) \in I$ means that attribute $m \in M$ of object $g \in G$ has values $w \in W$.

In the real world, most of the data is in the many-valued context. However, methods in the formal context analysis are only applied to the one-valued context of a formal context. Therefore, we need to transform the many-value context into a one-valued context. Scaling is a method to transform a many-valued context into a one-valued context.

A scaling transforms a many-valued context into a one-valued context by some scales which are also formal contexts. We call the one-valued context a derived context (Ganter & Wille, 1999; Wille, 2005).

**Definition 4.** *A scale for attribute* $m \in M$ *of a many-valued context* $(G,M,W,I)$ *is a one-valued context* ${S_m} = ({G_m},{M_m},{I_m})$ *with* ${G_m} \subseteq \{ w\mid (g,m,w) \in I,g \in G\}$ (Ganter & Wille, 1999; Wille, 2005).

A scale ${S_m} = ({G_m},{M_m},{I_m})$ interprets some values in ${G_m}$ of an attribute $m$ to some new attributes ${M_m}$. ${I_m}$ represents the interpretation.

**Definition 5.** *A derived context in the scaling of the many-valued context* $(G,M,W,I)$ *and scales* ${S_m}$ *for all* $m \in M$ is a context $(G,N,J)$ *where* (Wille, 2005)

(3) $$N = \bigcup\limits_{m \in M} {{M_m}}$$

*and for* $g \in G$ *and* $n \in N$:

(4) $$(g,n) \in J\;if\;and\;only\;if\;(g,m,w) \in I\;and\;(w,n) \in {I_m}$$

### Attribute implication and implicational base

An attribute implication over formal context $(G,M,I)$ is in the form $A \Rightarrow B$ where $A,B \subseteq M$. $A \Rightarrow B$ means that every object having all attributes in *A* has also all attributes in *B*. The attribute implication holds in the formal context if and only if each object respects it Ganter & Wille (1999). An object $g \in G$ respect the attribute implications if and only if the set of its attributes is a model of the attribute implication.

**Definition 6.** *Let* $A,B,T \subseteq M$. *T is a model of attribute implication* $A \Rightarrow B$ *if and only if* $A \not\subseteq T$ or $B \subseteq T$ (Ganter & Wille, 1999; Wille, 2005).

**Definition 7.** *An object* $g \in G$ *respects* $A \Rightarrow B$ over $(G,M,I)$ *if and only if* ${\{ g\} ^I}$ *is a model of the attribute implication* (Ganter & Wille, 1999; Wille, 2005).

An attribute implication holds a formal context $(G,M,I)$ if each object $g \in G$ respects the attribute implication.

Let ${\cal L}$ a set of attribute implications over a formal context $(G,M,I)$. We call ${\cal L}$ an implicational base of the formal context if the set is sound, complete, and non-redundant.

**Definition 8.** *A set of attribute implications* ${\cal L}$ *is an implicational base of formal context* $(G,M,I)$ *if the following holds:* (Ganter & Wille, 1999; Wille, 2005)

- *sound, if each attribute implication in* ${\cal L}$ *holds the formal context*
- *complete, if there is no attribute implication which holds the formal context, unless the attribute implication can be inferred from some attribute implications in* ${\cal L}$, *and*
- *non-redundant, if there is no attribute implication in* ${\cal L}$ *which can be inferred from the others in* ${\cal L}$.

### Implicational base of many-valued context

In many-valued context, we define the background-inferring problem which is whether an attribute implication holding in its derived-context is implied by the other ones holding also in the derived-context together with its scales.

**Definition 9.** *Let* ${\cal L}$ *a set of attributes implications which hold in the derived context from a many-valued context* $(G,M,W,I)$ *and scales* ${S_m}$ *for all* $m \in M$, ${\cal H}$ *information representing the scales, and* $A \Rightarrow B$ *an attribute implication which also holds in the derived context. The background-inferring problem is whether* (Hidayat, bin Ahmad & Ishak bin Desa, 2021):

(5) $${\cal L} \cup {\cal H} \models A \Rightarrow B$$

*It means that all models of* ${\cal L}$ *and* ${\cal H}$ *are also models of* $A \Rightarrow B$ (Ganter, 1996; Hidayat, bin Ahmad & Ishak bin Desa, 2021).

### Constraint

A constraint on a set of variables is a restriction on the values that they can take simultaneously. A constraint can be represented in many ways. However, a constraint can be represented as a set which contains all the legal compound labels for the subject variables (Tsang, 2014).

**Definition 10.** *Let W a finite set of variables and* ${D_x}$ *a domain of* $x \in W$. *A label in W is a pair* $< x,v \gt$ *where* $x \in W$ *and* $v \in {D_x}$, *which means that a value* $v$ *is assigned to a variable* $x$ (Tsang, 2014).

**Definition 11.** *Let* $\lt{x_i},{v_i} \gt$ *a label in W. A compound label over W is*

(6) $${L_W} = \left( {\lt{x_1},{v_1} > ,\lt{x_2},{v_2} > , \ldots ,\lt{x_n},{v_n} > } \right)$$

*which means that values* ${v_1}$, ${v_2}$, $\ldots$, ${v_n}$ *are assigned to variables* ${x_1}$, ${x_2}$, $\ldots$, ${x_n}$, *respectively* (Tsang, 2014).

**Definition 12.** *Let* $S = \{ {x_1},{x_2}, \ldots ,{x_n}\}$. *A constraint on set S, denoted by* ${C_s}$, *is a set of legal compound labels, where each compound label is in the form of* $\left( {\lt{x_1},{v_1} > ,\lt{x_2},{v_2} > , \ldots ,\lt{x_n},{v_n} > } \right)$ (Tsang, 2014).

**Definition 13.** *Let S and W finite sets. A compound label* ${L_W}$ *satisfies* ${C_S}$ *if there is a compound label* $L \in {C_S}$ *such that every pair* $< x,v \gt$ *in L is also a pair in* ${L_W}$ (Tsang, 2014).

## Methods

**Figure 2:**
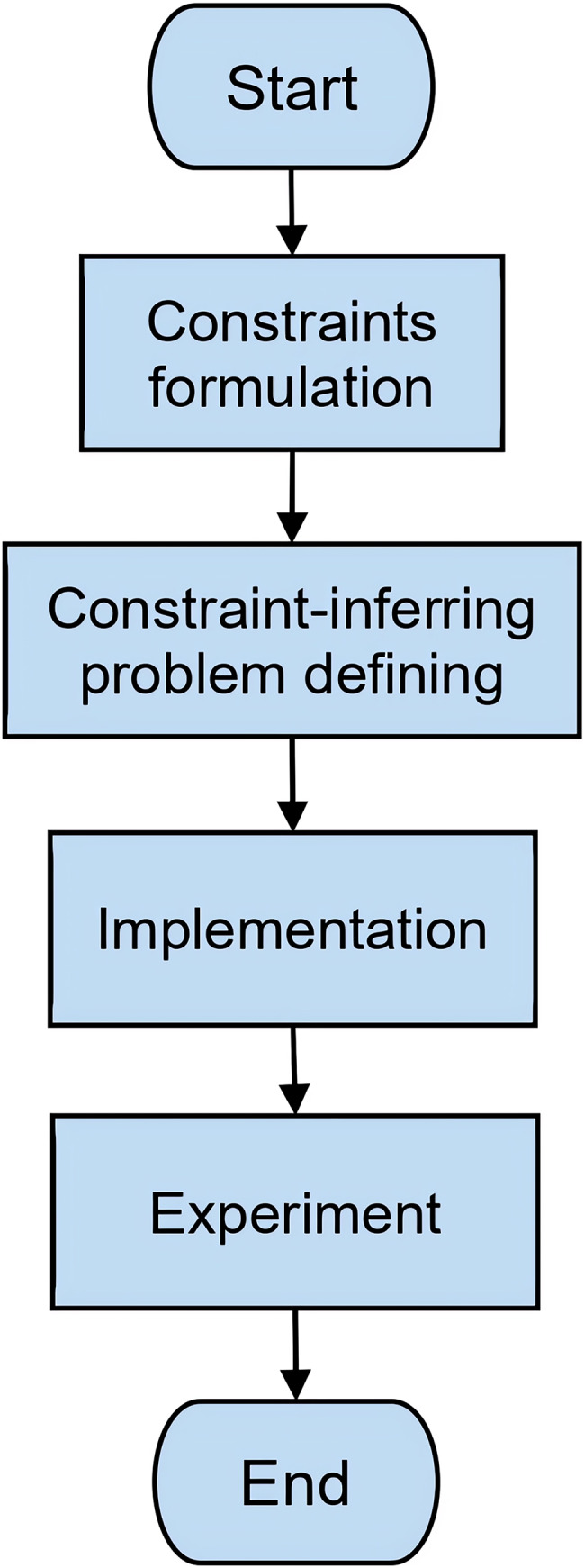
Research method.

Figure 2 shows steps of this research. Explanations of the steps are as follows:

1. Constraints formulation

In this step, we formalize constraints for a formal context mathematically. A constraint will be represented by a mathematical model. The model has to be concise such that it can represent all possible constraints for any formal contexts. Furthermore, we represents the model into a formal context.

Representation of constraints is needed in this research such that it can represent any real problem of constraints where a formal context satisfies. In addition, the representation will be used to define constraint-inferring problem which is next step of this research.

2. Constraint-inferring problem defining

In this step, we will define the constraint-inferring problem. The constraint-inferring problem is whether an attribute implication of implicational base of formal context can be inferred from the others together with some constraints which the formal constraint satisfies. In this step, we also propose an encoding of the problem into SAT problem. By the encoding, we can express the constraint-inferring problem into an equivalent SAT problem. We will solve the constraint-inferring problem by solving the SAT problem using SAT Solver.

Constraint-inferring problem definition and encoding into SAT problem is required by the proposed method which will generate a non-redundant implicational base. It will be implemented as a procedure to detect a redundant attribute implication, an attribute implication which can be inferred from the others together with constraints, such that the proposed method will ignore and remove the attribute implication from implicational base.

3. Implementation

We develop a method and a software code to generate a non-redundant implicational base of formal context with constraints. The main part of the software is to solve constraint-inferring problem for each attribute implication. For this purpose, the software will encode the problem into the SAT problem then solve it by a SAT solver.

Using this implementation, we will do experiments to prove that the proposed method is able to generate non-redundant implications correctly.

4. Experiment

We perform an experiment to generate a non-redundant implicational base of some formal contexts where there are some constraints which the formal context satisfies. This experiment also show how the proposed method is exactly able to remove all redundant attribute implications.

Preliminary result of this research had been presented in the IEEE 6th International Conference on Information Technology to obtain some comments and suggestions from scientific community. Thus, some portions of text in this article were previously published as a part of article presented in the conference (Hidayat, 2013). Part of this research which were presented in the conference article are formulation of constraints, defining of constraint-inferring problem, and encoding the constraint-inferring problem into the SAT problem. From the parts, we develop a method to generate a non-redundant implicational base, implement or code the method into Java programming language, and conduct experiment.

## Formal context with constraints

### Constraints for a formal context

Suppose we have a formal context $(G,M,I)$. We define a variable set $S = \{ {x_P}\mid P \subseteq M\}$ where the domain for each variable ${x_P}$ is ${D_P} = {2^P}$. Now we can give a constraint to restrict some attributes of $P \subseteq M$ for each object in *G*. A constraint can be written as follows:

(7) $${C_{\{ {x_P}\} }} = \{ (\lt{x_P},{v_P} > )\mid {v_P} \in D\}$$

where $D \subset {D_P}$ consisting allowed values to ${X_P}$.

**Example 1.** *Recall the formal context of “Bodies of Water” in* Fig. 1. *From the existing knowledge, there are some restrictions for some attributes. Attribute stagnant and attribute running, for example, have a restriction that each object absolutely has only one of both attributes. Objects tarn, lagoon, and lake, for instances, have attribute stagnant but do not have attribute running, whereas objects trickle, stream, and torrent, for instances, have attribute running but do not have attribute stagnant. Attribute inland and attribute maritime have a similar restriction, and also attribute constant and attribute temporary. Therefore, we have three constraints for the formal context*.

*Let:*

-
${P_1} = \{ stagnant,running\}$
-
${P_2} = \{ inland,maritime\}$, *and*
-
${P_3} = \{ constant,temporary\}$.

*Then, the constraints can be defined as follows:*

-
${C_{\{ {x_{{P_1}}}\} }} = \{ (\lt{x_{{P_1}}},\{ stagnant\} > ),(\lt{x_{{P_1}}},\{ running\} > )\}$
-
${C_{\{ {x_{{P_2}}}\} }} = \{ (\lt{x_{{P_2}}},\{ inland\} > ),(\lt{x_{{P_2}}},\{ maritime\} > )\}$
-
${C_{\{ {x_{{P_3}}}\} }} = \{ (\lt{x_{{P_3}}},\{ constant\} > ),(\lt{x_{{P_3}}},\{ temporary\} > )\}$

An object $g \in G$ satisfies a constraint ${C_{\{ {x_P}\} }}$ if the attributes combination belonging to $g$ in $P \subseteq M$ is a value assigned to ${x_P}$ in the constraint. For example, object $tarn$ satisfies three constraints in Example 1 since attributes combination belonging to the object in ${P_1}$, ${P_2}$, and ${P_3}$ are $\{ stagnant\}$, $\{ inland\}$ and $\{ constant\}$, which are assigned to ${x_{{P_1}}}$ in ${C_{\{ {x_{{P_1}}}\} }}$, ${x_{{P_2}}}$ in ${C_{\{ {x_{{P_2}}}\} }}$, and ${x_{{P_3}}}$ in ${C_{\{ {x_{{P_3}}}\} }}$, respectively.

**Definition 14.** *An object* $g \in G$ *of formal context* $(G,M,I)$ *satisfies a constraint* ${C_{\{ {x_P}\} }}$ *where* $P \subseteq M$ *if a compound label* ${L_{\{ {x_P}\} }} = (\lt{x_P},{\{ g\} ^I} \cap P > )$ *satisfies the constraint*.

**Definition 15.** *A formal context* $(G,M,I)$ *satisfies a constraint* ${C_{\{ {x_P}\} }}$ *if for all* $g \in G$, $g$ *satisfies the constraint*.

It is trivial to check that the formal context of “bodies of water” satisfies the three constraints in Example 1.

### Representing a constraint as a formal context

Interestingly, a constraint can be represented by a formal context. Let ${C_{\{ {x_P}\} }}$ a constraint of formal context $(G,M,I)$. The constraint can be represented as a formal context $({G_P},{M_P},{I_P})$ which is defined as follows:

-
${G_P} = {C_{\{ {x_P}\} }}$
-
${M_P} = P$
-
$(g,m) \in {I_P}$ for $g \in {G_p}$ and $m \in {M_P}$ if $g = (\lt{x_P},A > ) \in {C_{\{ {x_P}\} }}$ and $m \in A$

**Figure 3:**
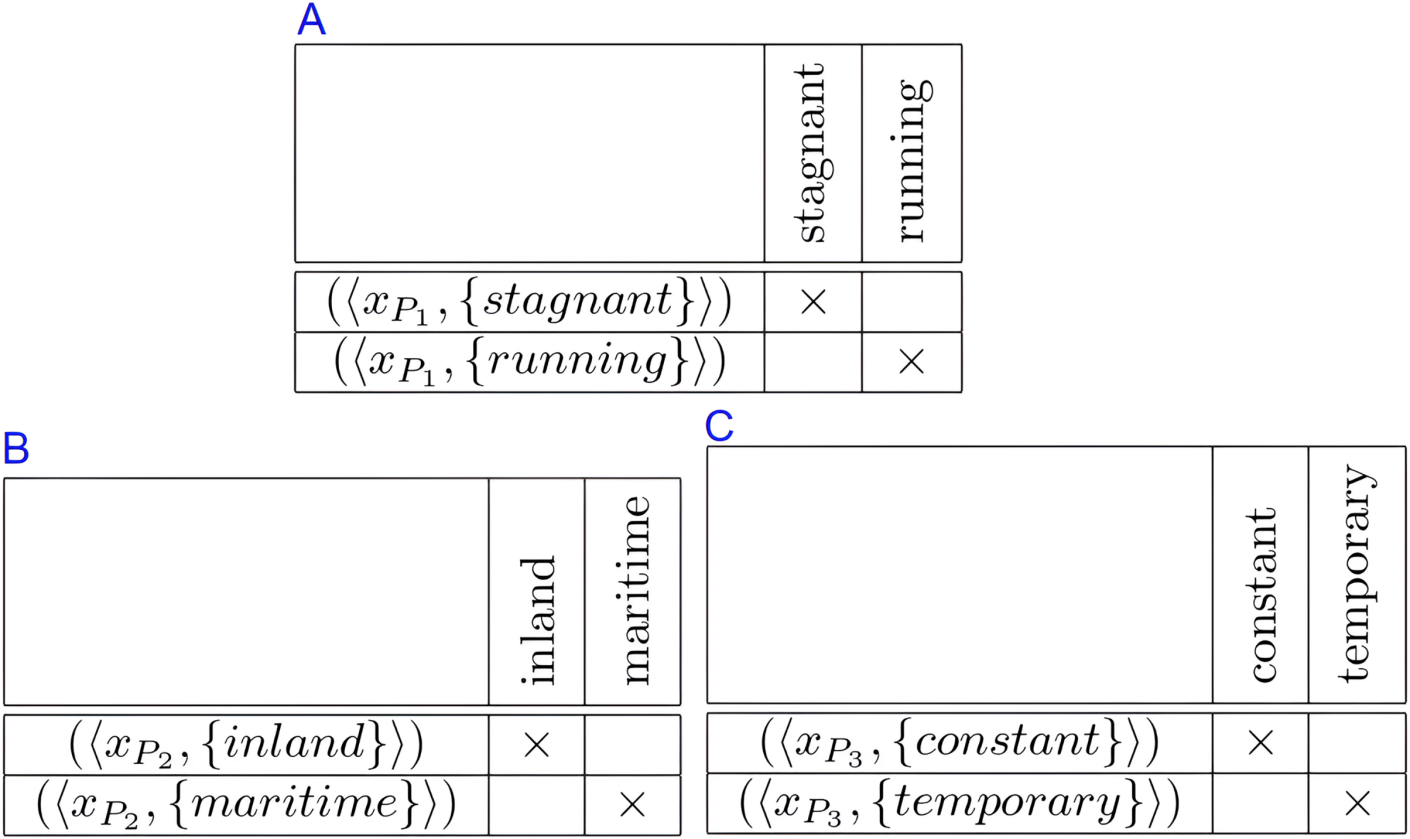
Formal contexts to represent constraints of formal context in Fig. 1: (A) constraint ${C_{\{ {x_{{P_1}}}\} }}$, (B) constraint ${C_{\{ {x_{{P_2}}}\} }}$, and (C) constraint ${C_{\{ {x_{{P_3}}}\} }}$.

**Example 2.** *Recall Example 1. The constraints* ${C_{\{ {x_{{P_1}}}\} }}$, ${C_{\{ {x_{{P_2}}}\} }}$, *and* ${C_{\{ {x_{{P_3}}}\} }}$ *are represented by the formal contexts in* Fig. 3.

By the representation, each object of the formal context representing a constraint is associated to a label of the constraint.

**Proposition 1.** *Let* $({G_P},{M_P},{I_P})$ *a representation of constraint* ${C_{\{ {x_P}\} }}$. *A formal context* $(G,M,I)$ *satisfies a constraint* $({G_P},{M_P},{I_P})$ *if for all* $g \in G$, *there is* ${g_P} \in {G_P}$ *such that* ${\{ g\} ^I} \cap {M_P} = {\{ {g_P}\} ^{{I_P}}}$.

*Proof*. First, we will prove that $(G,M,I)$ satisfies $({G_P},{M_P},{I_P})$ if for all $g \in G$, $g$ satisfies ${C_{\{ {x_P}\} }}$. Second, we will prove that for any $g \in G$, there is ${g_P} \in {G_P}$ such that ${\{ g\} ^I} \cap {M_P} = {\{ {g_P}\} ^{{I_P}}}$. The proof is as follows:

• (*G,M, I*) satisfies $({G_P},{M_P},{I_P})$

if $(G,M,I)$ satisfies ${C_{\{ {x_P}\} }}$

if for all $g \in G$, $g$ satisfies ${C_{\{ {x_P}\} }}$

• $g \in G$ satisfies ${C_{\{ {x_P}\} }}$

if a compound label ${L_{\{ {x_P}\} }} = (\lt{x_P},{\{ g\} ^I} \cap P > )$ satisfies ${C_{\{ {x_P}\} }}$

if there is a compound label $(\lt{x_P},A > ) \in {C_{\{ {x_P}\} }}$, such that ${\{ g\} ^I} \cap P = A$

if there is ${g_P} \in {G_P}$, which is associated to the compound label, such that ${\{ g\} ^I} \cap P = {\{ {g_P}\} ^{{I_P}}}$

if there is ${g_P} \in {G_P}$, such that ${\{ g\} ^I} \cap {M_P} = {\{ {g_P}\} ^{{I_P}}}$

## Constraint-inferring problem

Suppose there is a formal context and there are some constraints where the formal context satisfies. Let $A \Rightarrow B$ an attribute implication and ${\cal L}$ a set of attribute implications where all of the attribute implications hold in the formal context. Constraint-inferring problem is whether $A \Rightarrow B$ can be inferred from ${\cal L}$ and the constraints.

**Definition 16.** *Given an attribute implication* $A \Rightarrow B$, *which holds in a formal context* $(G,M,I)$, *a set of attribute implications* ${\cal L}$ *which also hold in the formal context, and* $n$ *constraints* ${C_{\{ {x_{{P_1}}}\} }},{C_{\{ {x_{{P_2}}}\} }}, \ldots ,{C_{\{ {x_{{P_n}}}\} }}$ *which the formal context satisfies. The constraint-inferring problem is whether:*

(8) $${\cal L} \cup {\cal R} \models A \Rightarrow B$$

*where* ${\cal R}$ *is a representation of the constraints* (Hidayat, 2013).

Refer to Eq. (5), expression in Eq. (8) also means that every model of ${\cal L} \cup {\cal R}$ is also model of $A \Rightarrow B$.

### Background-inferring problem is also constraint-inferring problem

If we make comparison between background-inferring problem and constraint-inferring problem, the difference is the information of ${\cal R}$ and ${\cal H}$. Fortunately, if both are similar, we can consider the constraint-inferring problem as the background-inferring problem. It will be proven that information of background in the background-inferring problem is also constraint.

**Proposition 2.** ${\cal H}$ *in the background-inferring problem is also information of constraints which the derived context satisfies* (Hidayat, 2013).

Proof. ${\cal H}$ in the background-inferring problem is information of scales. Thus, we will prove that scales are constraints which its derived-context satisfies.

Let $(G,N,J)$ a derived context of many-valued context $(G,M,W,I)$ and ${S_m} = ({G_m},{M_m},{I_m})$ a scale for an attribute $m \in M$. $(G,N,J)$ satisfies the constraint ${S_m} = ({G_m},{M_m},{I_m})$ if for all $g \in G$, there is ${g_m} \in {G_m}$ such that ${\{ g\} ^J} \cap {M_m} = {\{ {g_m}\} ^{{I_m}}}$ (Proposition 1).

Let $g \in G$ and $w \in W$ such that $(g,m,w) \in I$. By definition, we know that $w \in {G_m}$ and for all $n \in {M_m} \subseteq N$, $(g,n) \in J$ if $(w,n) \in {I_m}$. Thus, ${\{ g\} ^J} \cup {M_m} = {\{ w\} ^{{I_m}}}$.

Therefore, for all $g \in G$, there is always $w \in {G_m}$ where $(g,m,w) \in I$, such that ${\{ g\} ^J} \cap {M_m} = {\{ w\} ^{{I_m}}}$. Then, $(G,N,J)$ satisfies the constraint ${S_m} = ({G_m},{M_m},{I_m})$ (Hidayat, 2013).

### Encoding constraint-inferring problem into SAT problem

From Proposition 2, we can conclude that constraint-inferring problem is also background-inferring problem. Therefore the constraint-inferring problem can encod into a SAT problem using encoding of the background-inferring problem in Hidayat, bin Ahmad & Ishak bin Desa (2021) by replacing the information of background with the constraints as follows:

(9) $${\cal L} \cup {\cal R}\nvDash A \Rightarrow B$$

if and only if the following propositional formulae are satisfiable:

(10) $$\bigwedge\limits_{d\in D}\left( \bigwedge\limits_{c\in C}p_c\rightarrow p_d \right){\rm for\, each}\, C\Rightarrow D\in \cal L$$

(11) $$\bigvee\limits_{g\in G_p}\left(\left( \bigwedge\limits_{a\in {\{g\}^I}}p_a \right)\wedge \left( \bigwedge\limits_{a\in M_p{\backslash } {\{g\}^I}}\neg p_a \right) \right){\rm for\, each\, constraint}\, {(G_p,M_p,I_p)}\, \rm in\, \cal R$$

(12) $$\neg\left(\bigwedge\limits_{b\in B}\left(\bigwedge\limits_{a\in A}p_a\rightarrow {p_b}\right)\right)$$

where ${p_m}$ is a propositional variable corresponding to an attribute $m \in M$ of formal context $(G,M,I)$.

**Example 3.** *Recall Example 1 and Example 2. Suppose* ${\cal L}$ *consists of the following attribute implications:*

-
$\{ temporary\} \Rightarrow \{ natural,stagnant,inland\}$
-
$\{ maritime\} \Rightarrow \{ natural,stagnant,constant\}$

*and* ${\cal R}$ *is information of constraints* ${C_{\{ {x_{{P_1}}}\} }}$, ${C_{\{ {x_{{P_2}}}\} }}$, *and* ${C_{\{ {x_{{P_3}}}\} }}$. ${\cal L} \cup {\cal R}\nvDash \{running\} \Rightarrow \{ inland,constant\}$, *if only if the following formulae are satisfiable:*

$\eqalign{&({p_{temporary}} \to {p_{natural}}) \wedge ({p_{temporary}} \to {p_{stagnant}}) \wedge ({p_{temporary}} \to {p_{inland}})\\& ({p_{maritime}} \to {p_{natural}}) \wedge ({p_{maritime}} \to {p_{stagnant}}) \wedge ({p_{maritime}} \to {p_{constant}})\\& ({p_{stagnant}} \wedge \neg {p_{running}}) \vee (\neg {p_{stagnant}} \wedge {p_{running}})\\& ({p_{inland}} \wedge \neg {p_{maritime}}) \vee (\neg {p_{inland}} \wedge {p_{maritime}})\\& ({p_{constant}} \wedge \neg {p_{temporary}}) \vee (\neg {p_{constant}} \wedge {p_{temporary}})\\& \neg \left( {({p_{running}} \to {p_{inland}}) \wedge ({p_{running}} \to {p_{constant}})} \right)}$

## Non-redundant implicational base

In this section we present a proposed method to generate a non-redundant implicational base of formal context with some constraints where the formal context satisfies the constraints. We also present an implementation of the proposed method.

The proposed method is adopted from stem base algorithm (Ganter & Wille, 1999; Ganter & Obiedkov, 2016). Stem base algorithm is an algorithm to generate an implicational base of a formal context. Stem base algorithm is iterative process. Each iteration will generate an attribute implication which is sound and non-redundant based on all attribute implications generated in previous iteration. The algorithm ensures that after complete iteration, all generated attribute implications are complete.

The difference between the proposed method and the original algorithm is a decision whether a generated attribute implication in each iteration will be added into the implicational base or not. The attribute implication is added if and only if it can be inferred from all attribute implications generated in previous iteration together with the constraints (constraint-inferring problem).

**Figure 4:**
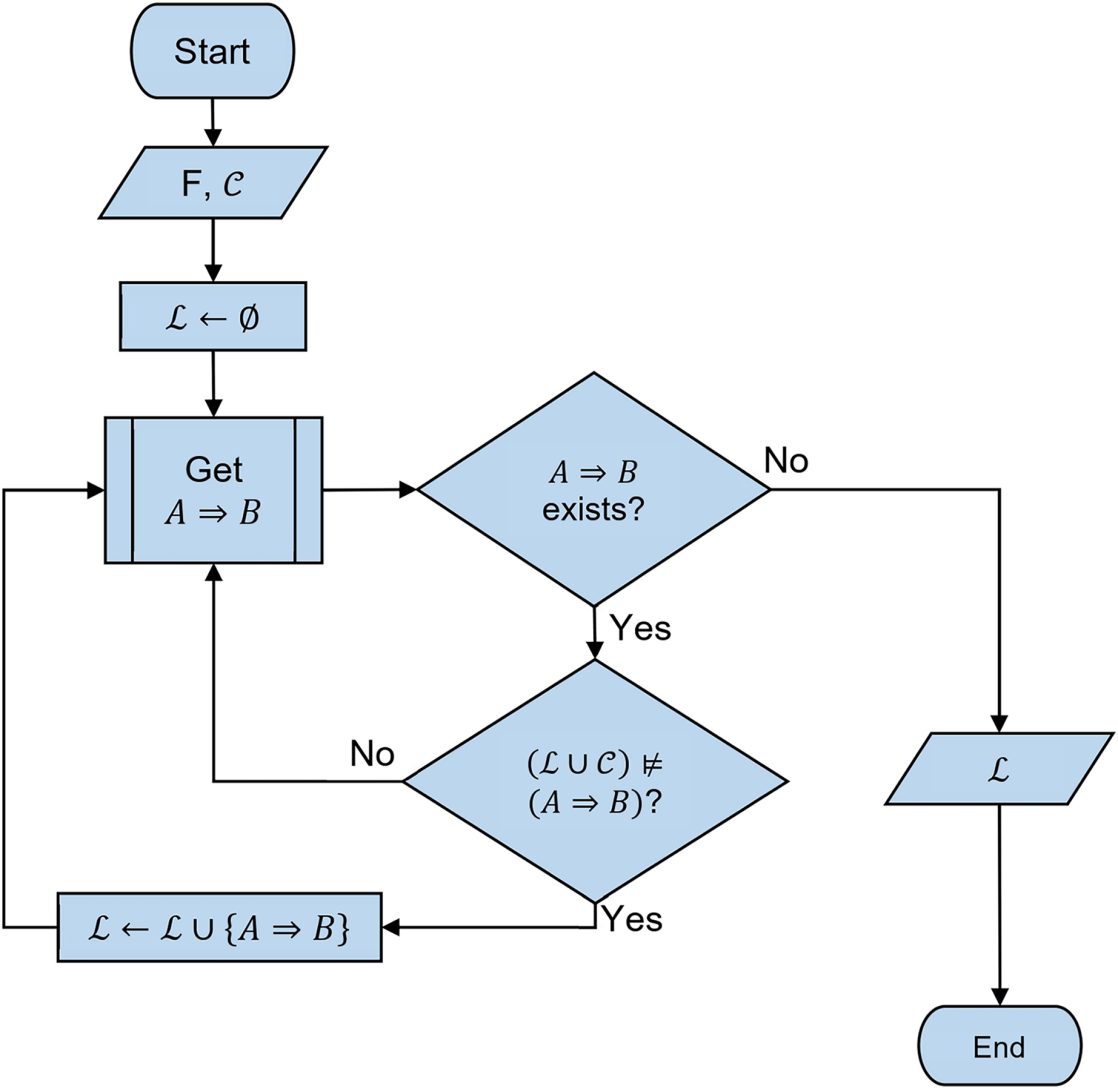
Flowchart of generating a non-redundant implicational base of formal context with constraints.

Figure 4 shows a flowchart to generate the non-redundant implicational base, where:

- *F* is a formal context
-
${\cal C}$ is a set of constraints
- “Get $A \Rightarrow B$” is a method to generate a new non-redundant and sound attribute-implication, which is adopted from stem-base algorithm.
-
${\cal L} \cup {\cal R} \nvDash A \Rightarrow B$ is negation of constraint-inferring problem.

The output of the flowchart is a set of attribute implication ${\cal L}$.

We implement the flowchart in Fig. 4 in Java Programming Language. We use the sat4j library (http://sat4j.org) as an SAT solver to solve an SAT problem. An SAT problem solved by the SAT solver has to be in conjunctive normal form (CNF). Thus, in this implementation we have to convert the SAT problem in Eqs. (10)–(12) into CNF. Therefore, the steps of solving a constraint-inferring problem in this implementation are as follows:

1) encoding the constraint-inferring problem into SAT problem in Eqs. (10)–(12)
2) converting the SAT problem into CNF
3) solving the SAT problem in CNF using sat4j.

## Experiment

Here we elaborate an experiment to show that the proposed method and its implementation work. In this experiment, we will generate non-redundant implicational base of some formal contexts with constraints using the implementation described in the previous section. The experimental results will be proved that they are correct.

### Experiment 1

In this experiment, we will generate non-redundant implicational base of formal context of “bodies of water” in Fig. 1 using the proposed method without and with constraints. The result will be compared with implicational base generated by stem base algorithm to see all removed attribute implications. And then, all removed attribute implications generated by proposed method will be proved that they are redundant based on corresponding constraints.

Recall the formal context in Example 1. Attributes of the formal context are $natural$, $artificial$, $stagnant$, $running$, $inland$, $maritime$, $constant$, $temporary$. From current knowledge, there are some constraints for the following attributes:

-
$stagnant$ and $running$
-
$inland$ and $maritime$
-
$constant$ and $temporary$

where constraints of each pair of the attributes are as follows that every object of the formal context has exactly one attribute of each pair. Let ${P_1} = \{ stagnant,running\}$, ${P_2} = \{ inland,maritime\}$, and ${P_3} = \{ constant,temporary\}$. The constraints can be expressed as follows:

-
${C_{\{ {x_{{P_1}}}\} }} = \{ (\lt{x_{{P_1}}},\{ stagnant\} ),(\lt{x_{{P_1}}},\{ running\} )\}$
-
${C_{\{ {x_{{P_2}}}\} }} = \{ (\lt{x_{{P_2}}},\{ inland\} ),(\lt{x_{{P_2}}},\{ maritime\} )\}$
-
${C_{\{ {x_{{P_3}}}\} }} = \{ (\lt{x_{{P_3}}},\{ constant\} ),(\lt{x_{{P_3}}},\{ temporary\} )\}$

Constraints ${C_{\{ {x_{{P_1}}}\} }}$, ${C_{\{ {x_{{P_2}}}\} }}$, and ${C_{\{ {x_{{P_3}}}\} }}$ are represented by formal context in Fig. 3.

The following are all attribute implications of implicational base of formal context using stem base algorithm:

1)
$\{ temporary\} \Rightarrow \{ natural,stagnant,inland\}$
2)
$\{ maritime\} \Rightarrow \{ natural,stagnant,constant\}$
3)
$\{ running\} \Rightarrow \{ inland,constant\}$
4)
$\{ stagnant,running,inland,constant\} \Rightarrow \{ natural,artificial,maritime,temporary\}$
5)
$\{ artificial\} \Rightarrow \{ inland,constant\}$
6)
$\{ natural,stagnant,inland,constant,temporary\} \Rightarrow \{ artificial,running,maritime\}$
7)
$\{ natural,stagnant,inland,maritime,constant\} \Rightarrow \{ artificial,running,temporary\}$
8)
$\{ natural,artificial,inland,constant\} \Rightarrow \{ stagnant,running,maritime,temporary\}$

The attribute implications will be referred to as ${r_1},{r_2},...,{\mkern 1mu} {\mathrm{and}}\,{\mkern 1mu} {r_{\mathrm{8}}}$, respectively.

Non-redundant implicational base will be generated by the proposed method with five cases as follows:

- without constraint (Case 1)
- with constraint ${C_{\{ {x_{{P_1}}}\} }}$ (Case 2)
- with constraint ${C_{\{ {x_{{P_2}}}\} }}$ (Case 3)
- with constraint ${C_{\{ {x_{{P_3}}}\} }}$ (Case 4)
- with constraint ${C_{\{ {x_{{P_1}}}\} }}$, ${C_{\{ {x_{{P_2}}}\} }}$, and ${C_{\{ {x_{{P_3}}}\} }}$ (Case 5)

Table 1 shows the attribute implications of implicational base of each case. First column is for implicational base generated by stem base algorithm and the others are for implicational base generated by proposed method for case 1, case 2,…, case 5, respectively. Each row represents the existence of each attribute implication of implicational base generated by stem base algorithm. Unchecked means that the attribute implication is considered as redundant and removed from implicational base. For example, attribute implication ${r_4}$, ${r_7}$, and ${r_6}$ are redundant attribute implications in implicational base of case 2, case 3, and case 4, respectively. Each row also refers to iteration of the proposed method in Fig. 4.

**Table 1:** Comparison of non-redundant implicational base with different constraints.

Attribute implication	Case 1	Case 2	Case 3	Case 4	Case 5
${r_1}$	$\checkmark$	$\checkmark$	$\checkmark$	$\checkmark$	$\checkmark$
${r_2}$	$\checkmark$	$\checkmark$	$\checkmark$	$\checkmark$	$\checkmark$
${r_3}$	$\checkmark$	$\checkmark$	$\checkmark$	$\checkmark$	
${r_4}$	$\checkmark$		$\checkmark$	$\checkmark$	
${r_5}$	$\checkmark$	$\checkmark$	$\checkmark$	$\checkmark$	$\checkmark$
${r_6}$	$\checkmark$	$\checkmark$	$\checkmark$		
${r_7}$	$\checkmark$	$\checkmark$		$\checkmark$	
${r_8}$	$\checkmark$	$\checkmark$	$\checkmark$	$\checkmark$	$\checkmark$

For case 1, the proposed method gives the same attribute implications generated by stem base algorithm. It proves that the proposed method gives the correct result. Regarding the other cases, the followings are proof of the redundant attribute implications for each case:

• Case 2: constraint ${C_{\{ {x_{{P_1}}}\} }}$

At iteration 4 (row 4), the generated attribute implication $A \Rightarrow B$ is

$\{ stagnant,running,inland,constant\} \Rightarrow \{ natural,artificial,maritime,temporary\}$

and ${\cal L} = \{ {r_1},{r_2},{r_3}\}$

Let *D* a model of ${\cal L} \cup \{ {C_{\{ {x_{{P_1}}}\} }}\}$. The followings are consequences:

$\to \{ stagnant,running\} \not\subseteq D$, because of constraint ${C_{\{ {x_{{P_1}}}\} }}$

$\to \{ stagnant,running,inland,constant\} \not\subseteq D$

$\to A \not\subseteq D$

$\to$ *D* is a model of $A \Rightarrow B$

Therefore, $A \Rightarrow B$ is redundant.

• Case 3: constraint ${C_{\{ {x_{{P_2}}}\} }}$

At iteration 7 (row 7), the generated attribute implication $A \Rightarrow B$ is

$\{ natural,stagnant,inland,maritime,constant\} \Rightarrow \{ artificial,running,temporary\}$

and ${\cal L} = \{ {r_1},{r_2},{r_3},{r_4},{r_5},{r_6}\}$.

Let *D* a model of ${\cal L} \cup \{ {C_{\{ {x_{{P_2}}}\} }}\}$. The followings are consequences:

$\to \{ inland,maritime\} \not\subseteq D$, because of constraint ${C_{\{ {x_{{P_2}}}\} }}$

$\to \{ natural,stagnant,inland,maritime,constant\} \not\subseteq D$

$\to A \not\subseteq D$

$\to$ *D* is a model of $A \Rightarrow B$

Therefore, $A \Rightarrow B$ is redundant.

• Case 4: constraint ${C_{\{ {x_{{P_3}}}\} }}$

At iteration 6 (row 6), the generated attribute implication $A \Rightarrow B$ is

$\{ natural,stagnant,inland,constant,temporary\} \Rightarrow \{ artificial,running,maritime\}$

and ${\cal L} = \{ {r_1},{r_2},{r_3},{r_4},{r_5}\}$.

Let *D* a model of ${\cal L} \cup \{ {C_{\{ {x_{{P_3}}}\} }}\}$. The followings are consequences:

$\to \{ constant,temporary\} \not\subseteq D$, because of constraint ${C_{\{ {x_{{P_3}}}\} }}$

$\to \{ natural,stagnant,inland,constant,temporary\} \not\subseteq D$

$\to A \not\subseteq D$

$\to$ *D* is a model of $A \Rightarrow B$

Therefore, $A \Rightarrow B$ is redundant.

• Case 5: constraint ${C_{\{ {x_{{P_1}}}\} }}$, ${C_{\{ {x_{{P_2}}}\} }}$, and ${C_{\{ {x_{{P_3}}}\} }}$

There are four redundant attribute implications as follows:

– $\{ running\} \Rightarrow \{ inland,constant\}$
– $\{ stagnant,running,inland,constant\} \Rightarrow \{ natural,artificial,maritime,temporary\}$
– $\{ natural,stagnant,inland,constant,temporary\} \Rightarrow \{ artificial,running,maritime\}$
– $\{ natural,stagnant,inland,maritime,constant\} \Rightarrow \{ artificial,running,temporary\}$

Last three attribute implications are same with redundant attribute implications in three previous cases. The proof are also same. Thus, only first attribute implication will be proved.

At iteration 3 (row), the generated attribute implication $A \Rightarrow B$ is:

$\{ running\} \Rightarrow \{ inland,constant\}$

and ${\cal L} = \{ {r_1},{r_2}\}$ or ${\cal L}$ contains the followings:

– $\{ temporary\} \Rightarrow \{ natural,stagnant,inland\}$ ( ${r_1}$)
– $\{ maritime\} \Rightarrow \{ natural,stagnant,constant\}$ ( ${r_2}$)

Let *D* a model of ${\cal L} \cup \{ {C_{\{ {x_{{P_1}}}\} }},{C_{\{ {x_{{P_2}}}\} }},{C_{\{ {x_{{P_3}}}\} }}\}$. Because of constraint ${C_{\{ {x_{{P_1}}}\} }}$, there are two possibilities of *D*:

– $\{ stagnant\} \subseteq D$
$\to \{ running\} \not\subseteq D$ because of ${C_{\{ {x_{{P_1}}}\} }}$
$\to A = \{ running\} \not\subseteq D$
$\to$ *D* is a model of $A \Rightarrow B$

– $\{ running\} \subseteq D$
Because of constraint ${C_{\{ {x_{{P_2}}}\} }}$, there are two possibilities of *D*:
  ∗ $\{ running,maritime\} \subseteq D$
$\to \{ running,maritime,natural,stagnant,constant\} \subseteq D$, because of ${r_2}$
$\to \{ running,stagnant\} \subseteq D$
$\to$ *D* contradicts constraint ${C_{\{ {x_{{P_1}}}\} }}$
$\to$ *D* is not a model of ${\cal L} \cup \{ {C_{\{ {x_{{P_1}}}\} }},{C_{\{ {x_{{P_2}}}\} }},{C_{\{ {x_{{P_3}}}\} }}\}$

∗ $\{ running,inland\} \subseteq D$
Because of constraint ${C_{\{ {x_{{P_3}}}\} }}$, there are also 2 possibilities of D:
  ⋅ $\{ running,inland,temporary\} \subseteq D$
$\to \{ running,inland,temporary,natural,stagnant\} \subseteq D$, because of ${r_1}$
$\to \{ running,stagnant\} \subseteq D$
$\to$ *D* contradicts constraint ${C_{\{ {x_{{P_1}}}\} }}$
$\to$ *D* is not a model of ${\cal L} \cup \{ {C_{\{ {x_{{P_1}}}\} }},{C_{\{ {x_{{P_2}}}\} }},{C_{\{ {x_{{P_3}}}\} }}\}$
  ⋅ $\{ running,inland,constant\} \subseteq D$
    Thus, $\{ running\} \subseteq D$ and *D* is a model of ${\cal L} \cup \{ {C_{\{ {x_{{P_1}}}\} }},{C_{\{ {x_{{P_2}}}\} }},{C_{\{ {x_{{P_3}}}\} }}\}$
$\to \{ running,inland,constant\} \subseteq D$
$\to B \subseteq \{ running,inland,constant\} \subseteq D$
$\to$ *D* is model of $A \Rightarrow B$

Therefore, $A \Rightarrow B$, which is $\{ running\} \Rightarrow \{ inland,constant\}$, is redundant.

### Experiment 2

In this experiment, we will show that the number of attribute implications in implicational base of formal context generated by the proposed method will decrease according to the number of constraints which the formal context satisfies. It happens because some attribute implications are redundant or can be inferred from the other attribute implications together with the constraint, such that they are ignored from implicational base.

In this experiment, the proposed method will generate implicational base of five formal contexts with corresponding constraints. The constraints are defined based on common existing knowledge or description of the formal context in data source. The formal contexts and constraints are as follows:

• Formal context of “living in water” (Wille, 1984)
Constraints for the following attributes:
  – ${P_1} = \{ dicotyledon,monocotyledon\}$
  – ${P_2} = \{ lives\;in\;water,lives\;on\;land\}$

• Formal context of “small natural number” (Ganter & Wille, 1999)
Constraints for the following attributes:
  – ${P_1} = \{ odd,even\}$
  – ${P_2} = \{ greater\;than\;2,greater\;than\;5\}$
  – ${P_3} = \{ prime,square\}$

• Formal context of “implicit information” (Fu, 2016)
Constraints for the following attributes:
  – ${P_1} = \{ Wastewater,Sludge\}$
  – ${P_2} = \{ Pressurised,Gravity\}$
  – ${P_3} = \{ Underground,Aboveground\}$

• Formal context of “object shapes” (Marín et al., 2021)
Constraints for the following attributes:
  – ${P_1} = \{ white,black\}$
  – ${P_2} = \{ large,small\}$
  – ${P_3} = \{ circle,square,triangle\}$

• Formal context of “failures event” (Rocco, Hernandez-Perdomo & Mun, 2020)
Constraints for the following attributes:
  – ${P_1} = \{ daytime,afternoon\}$
  – ${P_2} = \{ SERC,TRE,WECC\}$
  – ${P_3} = \{ Weather,Technical,Attack\}$

**Figure 5:**
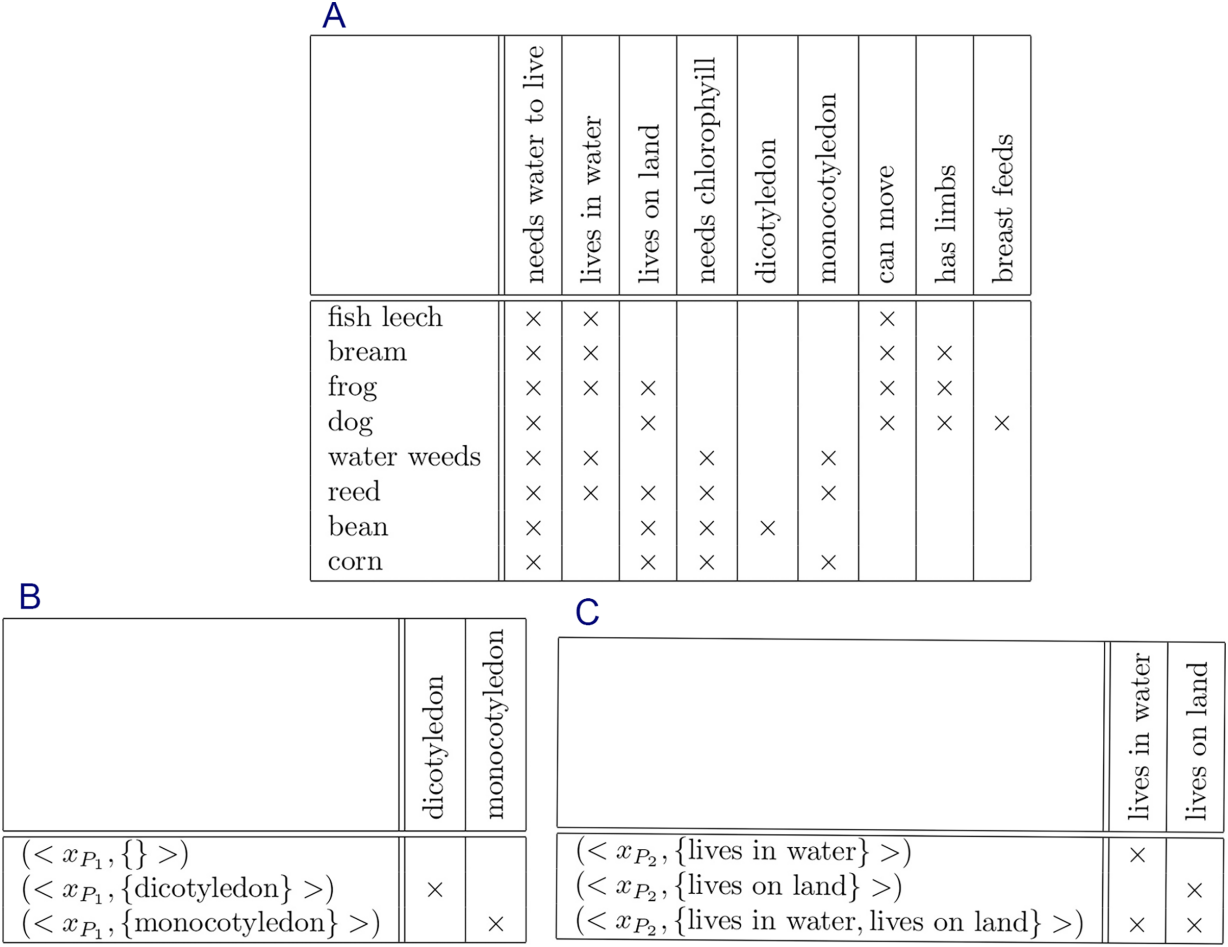
Formal context of “living in water” and its constraints: (A) formal context, (B) constraint for ${P_1} = \{ dicotyledon,monocotyledon\}$, and (C) constraint for ${P_2} = \{ lives in water,lives on land\}$.

**Figure 6:**
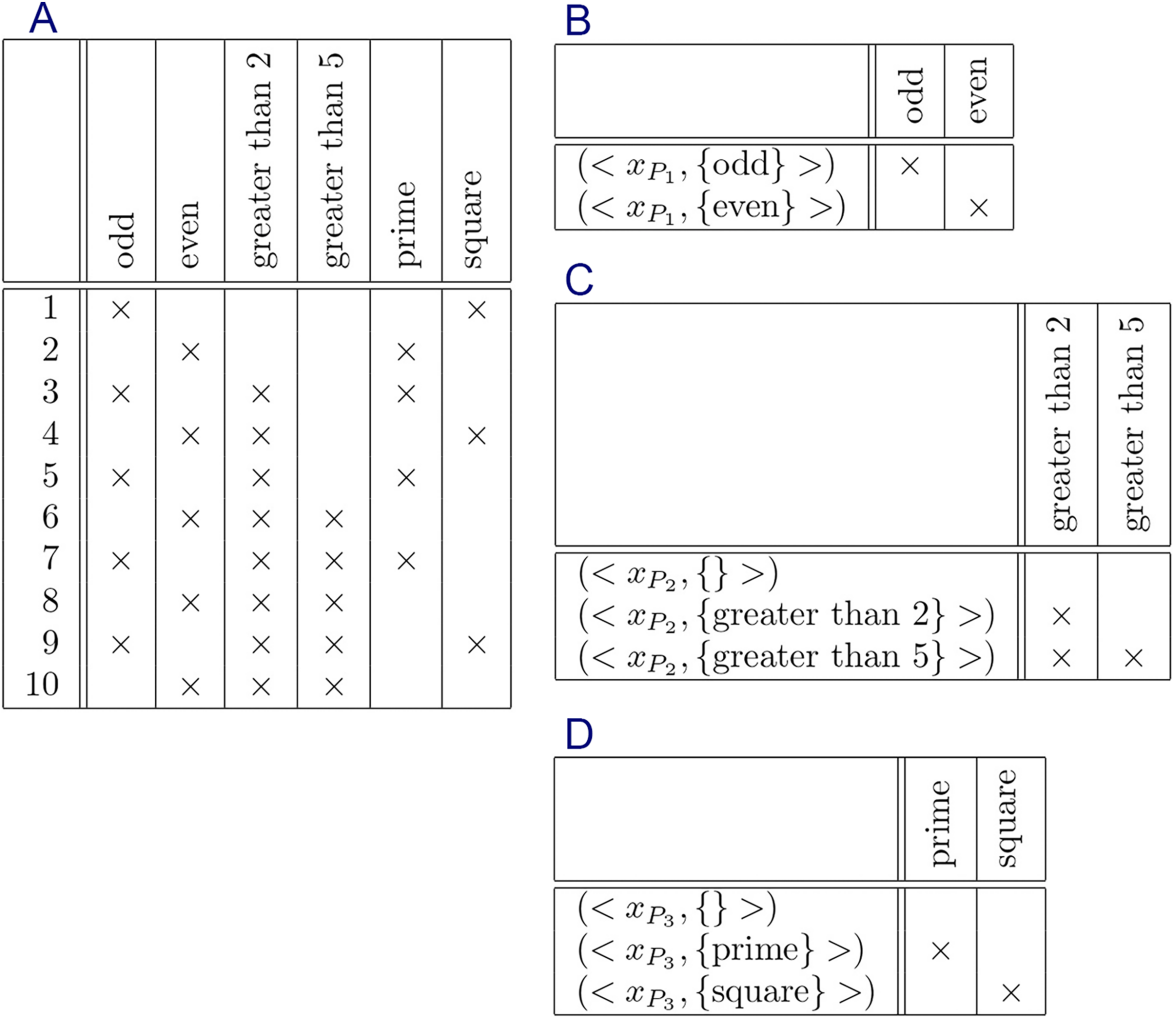
Formal context of “small natural number” and its constraints: (A) formal context, (B) constraint for ${P_1} = \{ odd,even\}$, (C) constraint for ${P_2} = \{ greater than 2,greater than 5\}$, and (D) constraint for ${P_3} = \{ prime,square\}$.

**Figure 7:**
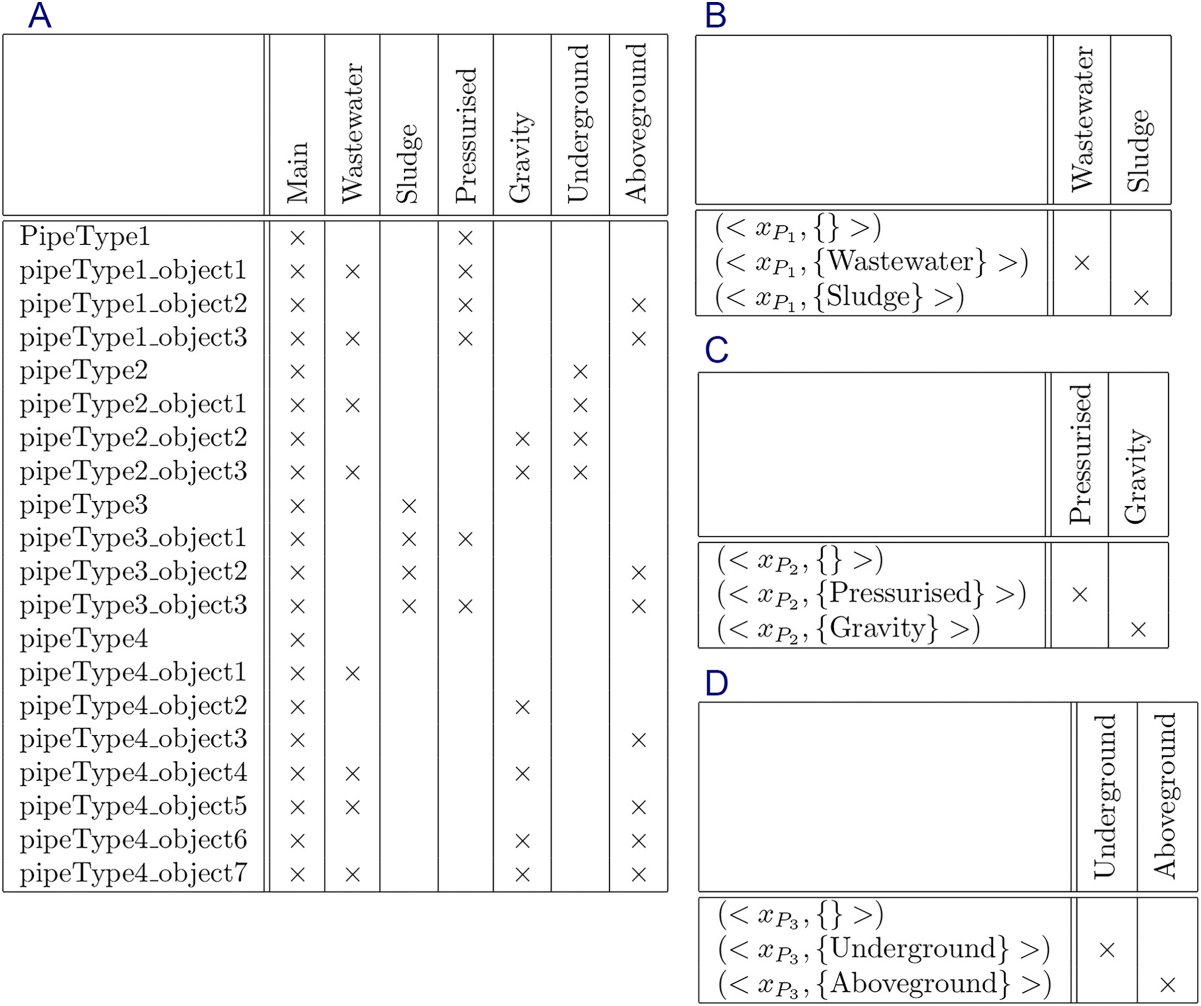
Formal context of “implicit information” and its constraints: (A) formal context, (B) constraint for ${P_1} = \{ Wastewater,Sludge\}$, (C) constraint for ${P_2} = \{ Pressurised,Gravity\}$, and (D) constraint for ${P_3} = \{ Underground$, $Aboveground\}$.

**Figure 8:**
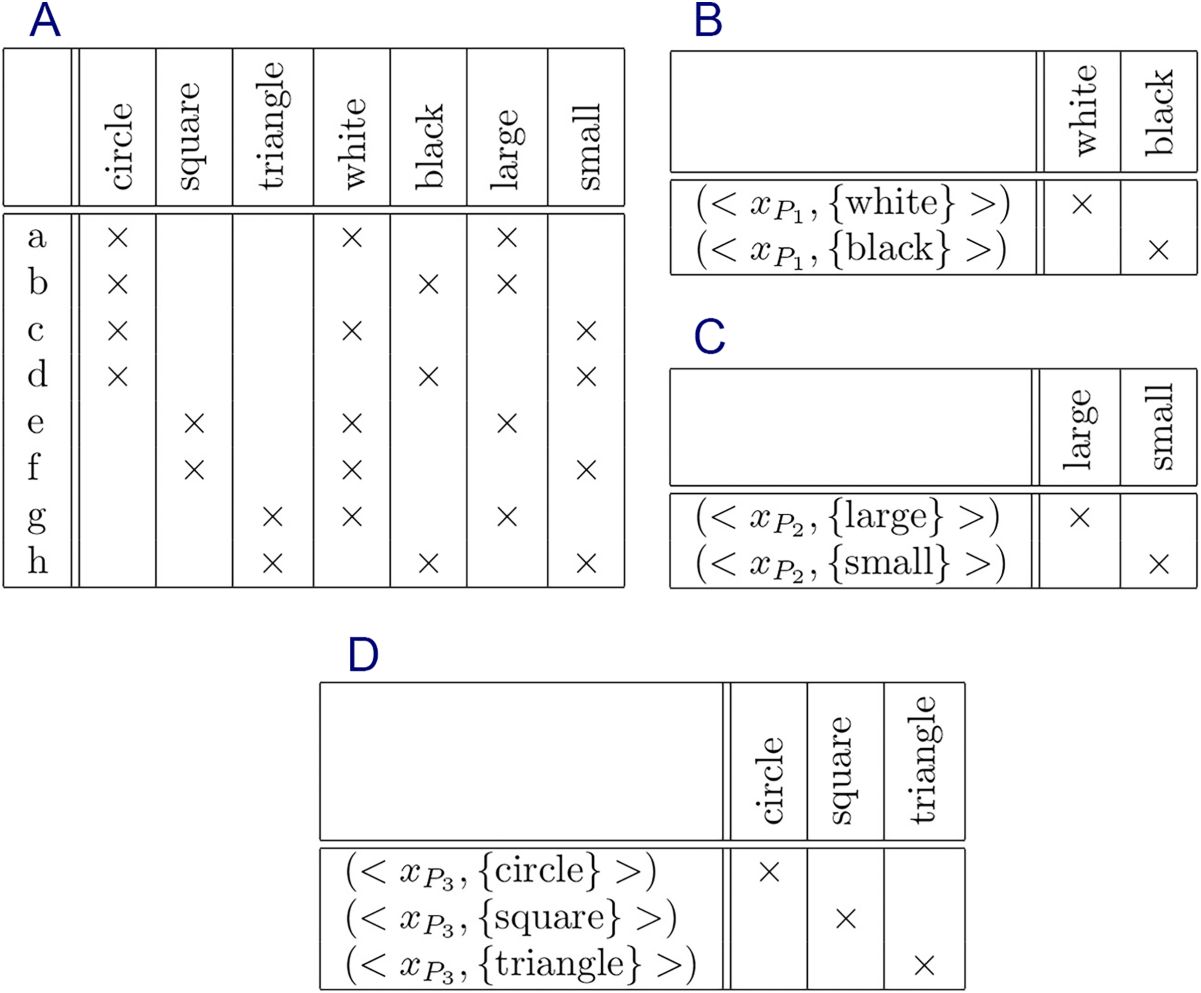
Formal context of “object shapes” and its constraints: (A) formal context, (B) constraint for ${P_1} = \{ white,black\}$, (C) constraint for ${P_2} = \{ large,small\}$, and (D) constraint for ${P_3} = \{ circle,square,triangle\}$.

**Figure 9:**
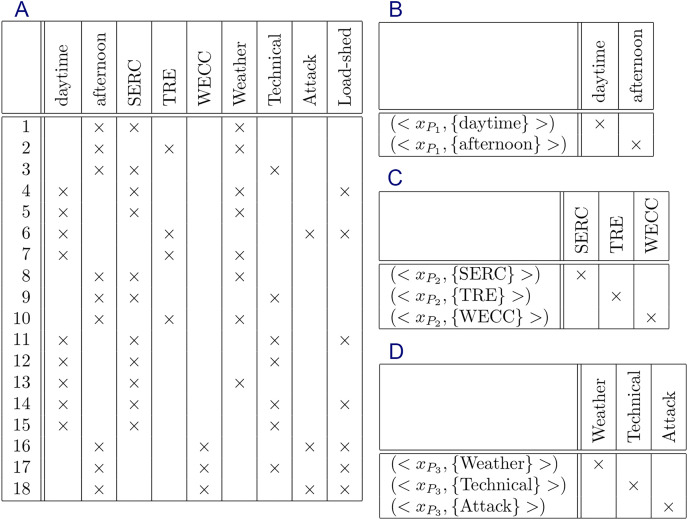
Formal context of “failures event” and its constraints: (A) formal context, (B) constraint for ${P_1} = \{ daytime,afternoon\}$, (C) constraint for ${P_2} = \{ SERC,TRE,WECC\}$, and (D) constraint for ${P_3} = \{ Weather,Technical,Attack\}$.

The formal context and their constraints are shown in Figs. 5–9, respectively.

For each formal context, some implicational bases are generated using stem base algorithm and the proposed method with no constraint, one constraint for ${P_1}$, two constraints for ${P_1}$ and ${P_2}$, and three constraints for ${P_1}$, ${P_2}$, and ${P_3}$. The number of attribute implications for each implicational base are presented by Table 2.

**Table 2:** Number of attribute implications of implicational base generated by stem base algorithm and the proposed method.

Formal context	Stem base algorithm	Proposed method			
		No constraint	One constraint	Two constraints	Three constraints
Living in water	11	11	10	9	
Small natural number	8	8	7	6	5
Implicit information	7	7	6	5	4
Object shapes	11	11	10	7	3
Failures event	15	15	13	10	7

**Figure 10:**
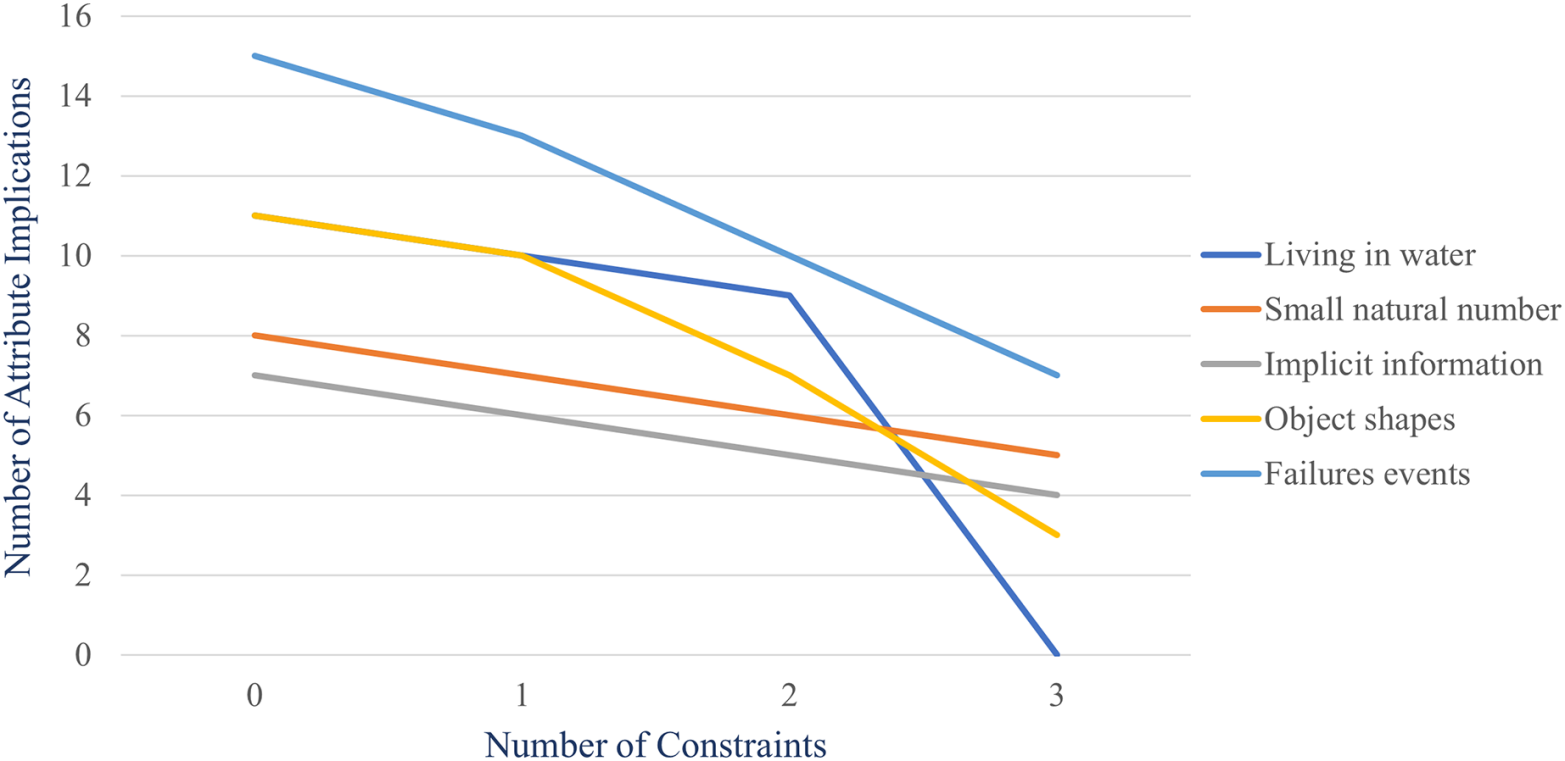
Number of attribute implications of implicational base generated by the proposed method in Experiment 2.

Table 2 shows that the number of attribute implications of implicational base decreases when the number of constraints increases. Figure 10 illustrates the reduction. According to design of this experiment, two-constraints case is adding constraint for ${P_2}$ to one-constraint one and three-constraints case is adding constraint for ${P_3}$ to two-constraints one. Thus, we can conclude that the more the constraints, the less the attribute implications of implicational base.

Therefore, the proposed method works properly. More constraints possibly imply more redundant attribute implications. The proposed method is able to check such attribute implications. It is proved by the result of this experiment.

### Experiment 3

In this experiment, we will apply the proposed method for large data. We select some datasets from UCI Machine Learning Repository. Specifically, we only focus on health datasets. Table 3 shows some health datasets from the repository for this experiment. Column instances and attributes represent number of instances and attributes, respectively.

**Table 3:** Health datasets from UCI machine learning repository.

Dataset	Instances	Attributes
Acute inflammations (Czerniak, 2009; Czerniak & Zarzycki, 2003)	120	8
Breast cancer Wisconsin (Wolberg, 1992; Wolberg & Mangasarian, 1990; Zhang, 1992)	699	10
Heart disease (Janosi et al., 1988; Detrano et al., 1989)	297	14
Healthy older people (Torres, Visvanathan & Ranasinghe, 2016; Torres et al., 2013)	231	10
Hepatitis C virus (HCV) (Lichtinghagen, Klawonn & Hoffman, 2020; Hoffmann et al., 2018; Lichtinghagen et al., 2013)	589	13
Audiology (UCI, 1992; Bareiss, Porter & Wier, 1988)	200	22
Autistic spectrum disorder screening data for adolescent (Tabtah, 2017, Thabtah, 2017b)	104	21
Autistic spectrum disorder screening data for children (Thabtah, 2017a, 2017b)	292	21
Breast cancer (Zwitter & Soklic, 1988a; Michalski et al., 1986)	277	10
Breast tissue (S & Jossinet, 2010; Jossinet, 1996; da Silva, de Sá & Jossinet, 2006)	106	10
Caesarian section classification (Amin & Ali, 2018, 2017)	80	6
Cervical cancer (Risk Factors) (Fernandes, Cardoso & Fernandes, 2017b, 2017a)	858	36
Contraceptive method choice (Lim, 1997; Lim, Loh & Shih, 2000)	1,473	10
Cryotherapy (Khozeimeh et al., 2018, 2017a, 2017b)	90	7
Diabetic retinopathy debrecen (Antal & Hajdu, 2014a, 2014b)	1,151	20
EEG eye state (Roesler, 2013)	14,980	15
Exasens (UCI, 2020a; Zarrin, Roeckendorf & Wenger, 2020)	100	8
Fertility (Gil & Girela, 2013; Méndez et al., 2012)	100	10
Heart failure clinical records (UCI, 2020b; Chicco & Jurman, 2020)	299	13
Hepatitis (UCI, 1988; Diaconis & Efron, 1983; Cestnik, Kononenko & Bratko, 1987a)	135	17
Liver disorders (UCI, 1990; McDermott & Forsyth, 2016)	345	6
Lung cancer (Hong & Yang, 1992; Hong & Yang, 1991)	32	56
Lymphography (Zwitter & Soklic, 1988b; Cestnik, Kononenko & Bratko, 1987b; Clark & Niblett, 1987; Michalski et al., 1986)	148	19
Mammographic mass (Elter, 2007)	830	46
Post-operative patient (Summers & Woolery, 1993; Woolery et al., 1991)	87	9
Primary tumor (Zwitter & Soklic, 1988c; Cestnik, Kononenko & Bratko, 1987b; Clark & Niblett, 1987; Michalski et al., 1986)	164	16
SPECT heart (Cios & Lukasz Kurgan, 2001)	267	23
Statlog (Heart) (UCI, 1999)	270	14
Thyroid disease (Quinlan, 1987)	8,861	23

A formal context will be created from each dataset. An instance of dataset becomes an object of the formal context whereas an attribute of dataset becomes some attributes of the formal context. The number of attributes of formal context depends on the interpretation of each value of each attribute in the dataset. To focus on generating the non-redundant implicational base, the creation process of each formal context is not explained in this article. However, we show the important information of the formal context in Table 4. Column attributes represent the number of attributes of formal context. Certainly, the number differs with the number of attributes of corresponding dataset.

**Table 4:** Formal context of health datasets.

Formal context	Objects	Attributes	Class attributes
Acute inflammations	120	19	4
Breast cancer Wisconsin	699	38	2
Heart disease	297	46	2
Healthy older people	231	33	4
Hepatitis C virus (HCV)	589	42	5
Audiology	200	185	24
Autistic spectrum disorder screening data for adolescent	104	87	2
Autistic spectrum disorder screening data for children	292	104	2
Breast cancer	277	43	2
Breast tissue	106	39	6
Caesarian section classification	80	18	2
Cervical cancer (Risk Factors)	858	107	2
Contraceptive method choice	1,473	29	3
Cryotherapy	90	25	2
Diabetic retinopathy debrecen	1,151	71	2
EEG eye state	14,980	72	2
Exasens	100	26	3
Fertility	100	30	2
Heart failure clinical records	299	36	2
Hepatitis	135	38	2
Liver disorders	345	18	3
Lung cancer	32	223	3
Lymphography	148	66	4
Mammographic mass	830	24	2
Post-operative patient	87	27	3
Primary tumor	164	39	6
SPECT heart	267	46	2
Statlog (Heart)	270	38	2
Thyroid disease	8,861	52	6

Table 4 also shows the number of class attributes (column ‘class attributes’). Class attributes are attributes of formal context as interpretation of class attributes of dataset. As classification data which is described in UCI repository, each dataset in this experiment has one class attribute or more. A value in class attribute of a dataset refers to a class of an instance. To maintain the class information, a class attribute in formal context corresponds to a class in class attribute of corresponding dataset. For example, Audiology dataset has a class attribute whose name is class. The class attribute has 24 possible values which means that there are 24 classes of instances. Therefore, there are 24 class attributes in the formal context of Audiology.

Because of some class attributes, there will be a constraint of the attributes. The constraint is that each object of formal context will have exactly one of the class attributes. For example, there are class attributes of formal context of “Healthy older people” where the class attributes are *Label of activity: sit on bed*, *Label of activity: sit on chair*, *Label of activity: lying*, and *Label of activity: ambulating*, which correspond to class attribute of “Label of activity” in the “Healthy older people” dataset. Undoubtedly, each object in this formal context will have exactly one of the attributes.

Thus, in general, each formal context in this experiment has constraints on class attributes where each constraint is related to a class attribute of corresponding dataset. Let ${P_a} = \{ {a_1},{a_2}, \ldots ,{a_k}\} \subseteq M$ a set of class attributes of formal context $(G,M,I)$ where ${P_a}$ is related to a class attribute $a$ of corresponding dataset. Then, there is a constraint for ${P_a}$ as follows:

${C_{\{ {x_{{P_a}}}\} }} = \{ (\lt{x_{{P_a}}},\{ {a_1}\} ),(\lt{x_{{P_a}}},\{ {a_2}\} ), \ldots ,(\lt{x_{{P_a}}},\{ {a_k}\} )\}$

Let $a$ class attribute “Label of activity” in the “Healthy older people” dataset. The formal context of “Healthy older people” has the following constraint:

${C_{\{ {x_{{P_a}}}\} }} = \left\{ {\matrix{ {(\lt{x_{{P_a}}},\{ Label of activity: sit on bed\} ),(\lt{x_{{P_a}}},\{ Label of activity: sit on chair\} ),} \cr  {(\lt{x_{{P_a}}},\{ Label of activity: lying\} ),(\lt{x_{{P_a}}},\{ Label of activity: ambulating\} )} \cr  } } \right\}$

Fortunately, all formal contexts in this experiment have one constraint only, except formal context of “Acute Inflammation”. The formal context has two constraints.

Therefore, in this experiment we generate a non-redundant implicational base of each formal context created from health datasets using the proposed method where constraints are on class attributes of the formal context. As comparable, we also generate an implicational base of same formal context using stembase algorithm which do not consider any constraints. Table 5 shows number of attribute implications of both implicational bases as the result of this experiment. The table also shows number of redundant attribute implications which is the difference between both. Percentage of redundancy in the table is ratio of the number of redundant attribute implications to the number of attribute implications without constraint.

**Table 5:** Implicational base of formal context of health datasets.

Formal context	Number of attribute implications			Percentage of redundancy
	Without constraints	With constraints	Redundant	
Acute inflammations	69	43	26	38%
Breast cancer Wisconsin	713	637	76	11%
Heart disease	4,494	3,300	1,194	27%
Healthy older people	122	103	19	16%
Hepatitis C virus (HCV)	1,453	1,378	75	5%
Audiology	1,691	1,349	342	20%
Autistic spectrum disorder screening data for adolescent	124	123	1	1%
Autistic spectrum disorder screening Data for children	2,130	1,909	221	10%
Breast cancer	3,300	3,129	171	5%
Breast tissue	122	98	24	20%
Caesarian section classification	73	61	12	16%
Cervical cancer (Risk Factors)	700	646	54	8%
Contraceptive method choice	1,529	1,221	308	20%
Cryotherapy	225	175	50	22%
Diabetic retinopathy debrecen	7,297	7,046	251	3%
EEG eye state	81	42	39	48%
Exasens	52	46	6	12%
Fertility	681	567	114	17%
Heart failure clinical records	5,183	3,605	1,578	30%
Hepatitis	3,096	2,920	176	6%
Liver disorders	52	48	4	8%
Lung cancer	361	357	4	1%
Lymphography	251	245	6	2%
Mammographic mass	277	218	59	21%
Post-operative patient	522	391	131	25%
Primary tumor	2,492	2,294	198	8%
SPECT heart	2,290	2,199	91	4%
Statlog (Heart)	5,029	3,563	1,466	29%
Thyroid disease	3,215	2,727	488	15%

**Figure 11:**
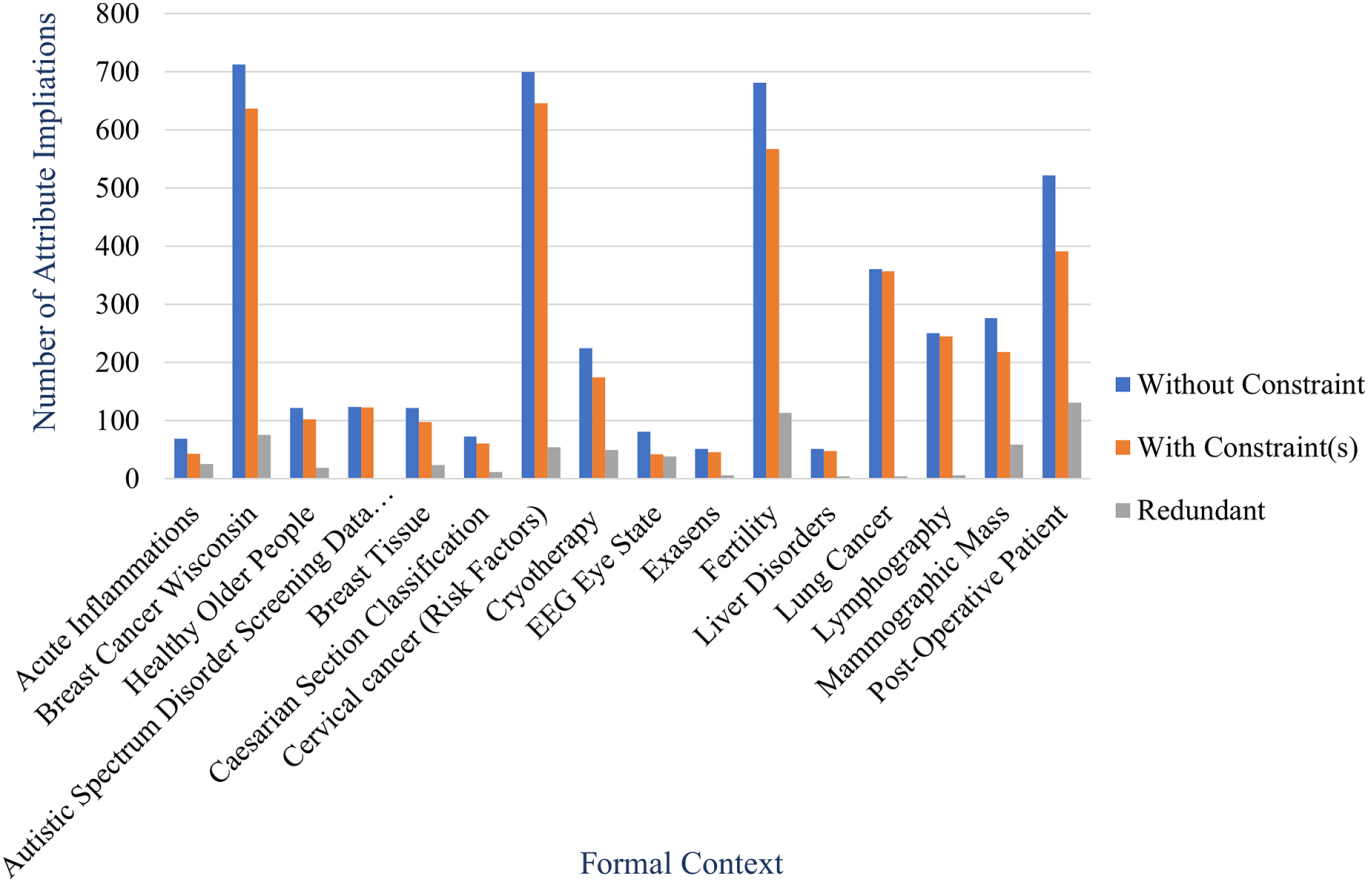
Number of attribute implications of implicational base in Experiment 3 where the number of attribute implication without constraint is less than 1,000.

**Figure 12:**
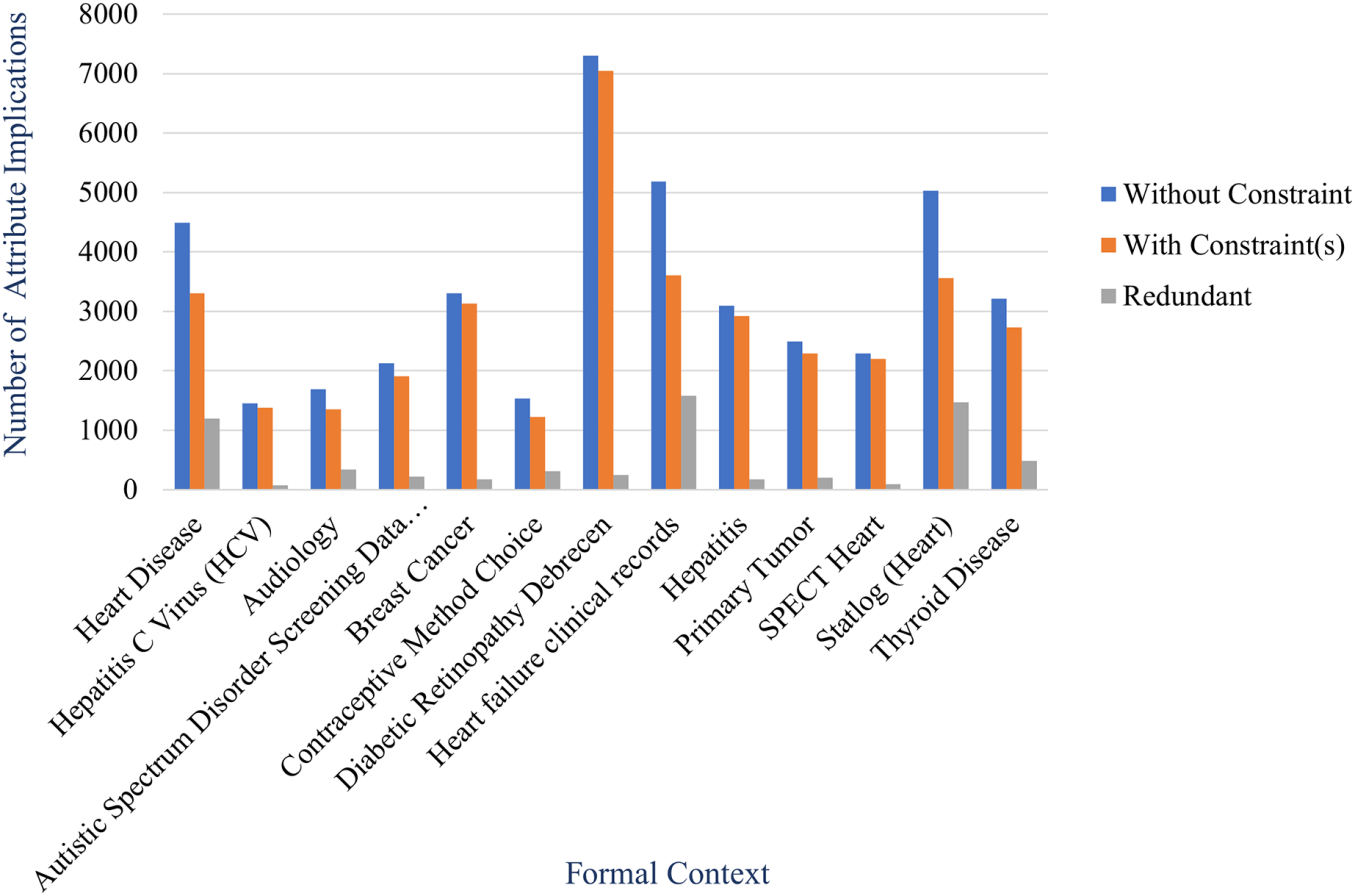
Number of attribute implications of implicational base in Experiment 3 where the number of attribute implication without constraint is more than 1,000.

**Figure 13:**
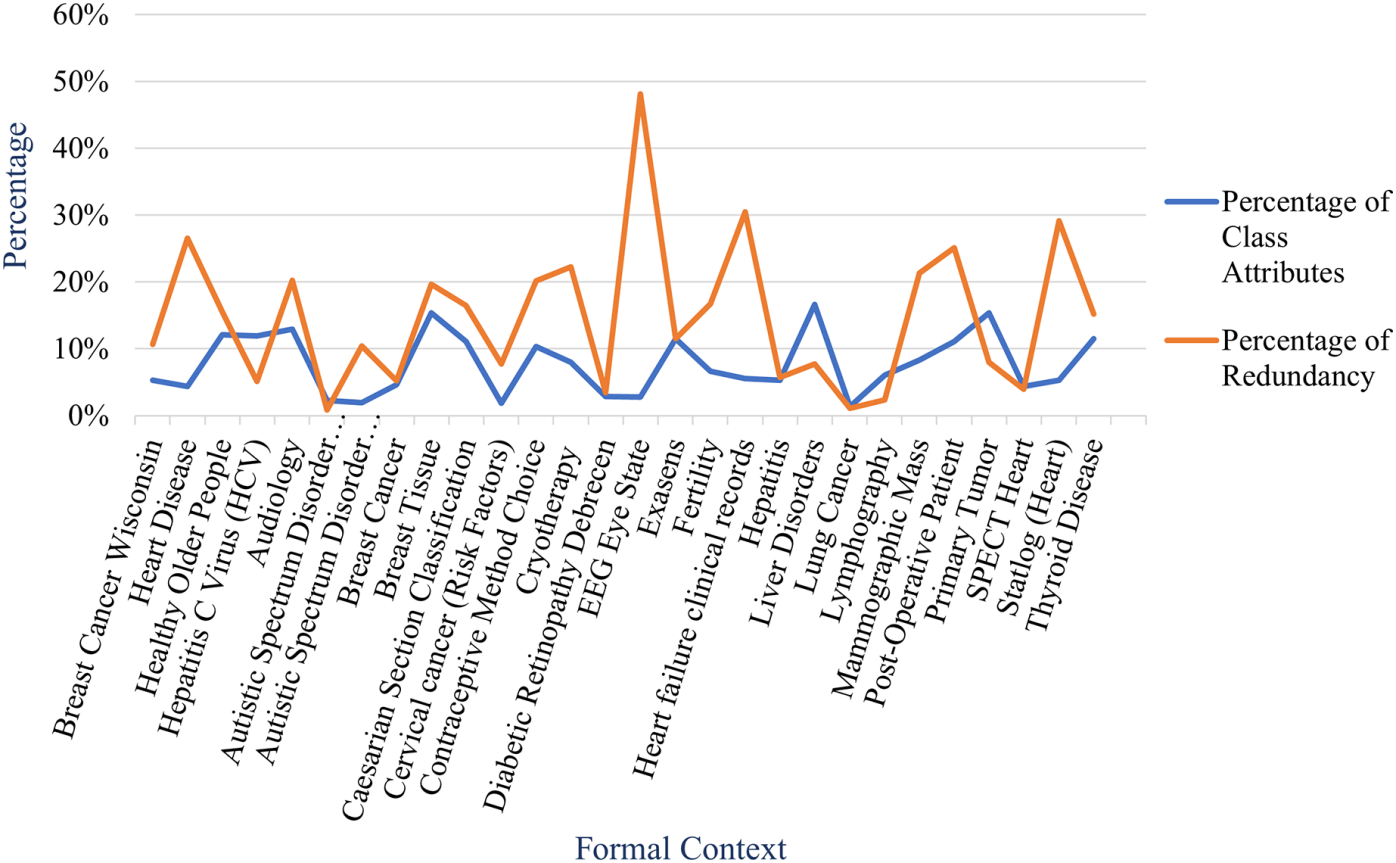
Comparison between percentage of redundant attribute implications and percentage of class attributes of formal contexts in Experiment 3.

From the table, we obtain that the proposed method can reduce the number of attribute implications of implicational base effectively. The reduction shown in the table is described more clearly in Figs. 11 and 12 by comparing the number of attribute implications without constraint (generated by stem base algorithm) and the number of attribute implications with constraint(s) (generated by proposed method). The figures also inform us that the reduction of each formal context varies even the reduction is expressed by the percentage of redundancy as described in Fig. 13.

Let percentage of class attributes a ratio of number of class attributes to number of attributes for a formal context, Fig. 13 also shows that there is no correlation between percentage of redundancy and percentage of class attributes. More precisely, the correlation coefficient of both is 0.240. Moreover, there is also no correlation between the percentage of redundancy and either number of objects, number of attributes, or number of class attributes. Table 6 presents correlation coefficients between the percentage of redundancy and the parameters of formal context.

**Table 6:** Correlation coefficient between each pair of number of objects, number of attributes, number of class attributes, percentage of class attributes, and percentage of redundancy.

	Number of objects	Number of attributes	Number of class attributes	Percentage of class attributes	Percentage of redundancy
Number of objects	1				
Number of attributes	0.040	1			
Number of class attributes	−0.026	0.470	1		
Percentage of class attributes	−0.132	−0.395	0.380	1	
Percentage of redundancy	0.461	−0.258	0.047	0.240	1

Therefore, reduction of number of attribute implications depends on redundancy of some attribute implications with others in implicational base of formal context together with constraints which the formal context satisfies. It implies that the proposed method can check redundant attribute implications and remove them from implicational base to obtain the high quality of generated knowledge.

### Summary of experiment

In Experiment 1, we prove that the method proposed in this research is successful to check all redundant attribute implications and ignore them such that it can generate a non-redundant implicational base of formal context. Every ignored attribute implication has been proved that it is redundant. We also show which constraints cause the redundancy since these redundancies are caused by constraints which the formal context satisfies.

In the experiments also, more specifically in Experiment 2, we show that more constraints imply more attribute implications ignored by the proposed method. Since constraints are our existing knowledge regarding to the formal context, more constraints we know mean more attribute implications actually representing our knowledge. Consequently, more attribute implications are redundant and will be ignored. Therefore, the proposed method is effective to generate a non-redundant implicational base of formal context with constraint.

In addition, in Experiment 3 we also show that the proposed method successfully works for formal contexts from large data. The proposed method can reduce the number of attribute implications of implicational base if there are one or more constraints which the formal context satisfies. Since the proposed method has been proved that it gives a correct result in Experiment 1, we assume that the proposed method can also check and ignore redundant attribute implications of implicational base of the large formal contexts. Consequently, the proposed method is also effective to generate a non-redundant implicational base of large formal context.

Last but not least, the proposed method can generate an implicational base in higher quality than one generated by the stembase algorithm which is mainstream algorithm in generating an implicational base in formal context analysis. The higher quality is achieved by ignoring all redundant attribute implications which can be inferred from constraints as existing knowledge. To support the conclusion, in these experiments we generate implicational base using the proposed method and the algorithm for same formal context and then compare both implicational bases.

## Conclusion and future works

We successfully proposed a method to generate a non-redundant implicational base of formal context with information of constraints which the formal context satisfies. This method will ignore some redundant attribute implications and remove them from the implicational base. A redundant attribute implication is attribute implication which can be inferred from the others together with the constraints. This method will improve the quality of knowledge generated by formal concept analysis, in this case is implicational base, because the constraints are a prior knowledge which is already known. Therefore, the generated knowledge is really new as expected in knowledge discovery. This will intensify the role of formal context analysis in this area.

In this article, we successfully formalized a mathematical model of constraints of formal context. This model is able to represent any constraints since it enumerates all possible values as restrictions of attributes of a formal context. Using this model, a problem to check whether an attribute implication is redundant based on constraints can be defined and encoded into a SAT problem. Thus, the proposed method can be applied to any constraints of formal context.

We also successfully defined the redundancy of an attribute implication as constraint-inferring problem. In this article, we proposed an encoding the problem into a SAT problem. With this encoding we can solve the problem using the SAT solver. After implementation of the proposed method, we successfully conducted experiments to show that the proposed method is able to generate the non-redundant implicational base using this encoding.

Some experiments with real data of formal context with constraints are be implemented in our next research. From these experiments we will show that we can reduce the size and also improve the quality of implicational base by ignoring some redundant attribute implications which can be inferred from others in the implicational base together with the constraints.

## Supplemental Information

10.7717/peerj-cs.1806/supp-1 Supplemental Information 1 Generating a non-redundant implicational base of formal context with constraints. The code is in Java Programming Language. It requires SAT4J Core library (available at http://www.sat4j.org/maven234/org.ow2.sat4j.core/index.html).
This Java implementation is for generating a non-redundant implicational base of a formal context which is restricted by some constraints. It includes an implementation for encoding a constraint-inferring problem into a SAT problem and solving the SAT problem using a SAT solver.

## Additional Information and Declarations

### Data Availability

The following information was supplied regarding data availability: The code is available in the Supplemental File.
